# The factor structure of the Edinburgh Postnatal Depression Scale among perinatal high-risk and community samples in London

**DOI:** 10.1007/s00737-021-01153-0

**Published:** 2021-07-10

**Authors:** Alexandra Lautarescu, Suresh Victor, Alex Lau-Zhu, Serena J. Counsell, A. David Edwards, Michael C. Craig

**Affiliations:** 1 Centre for the Developing Brain, School of Biomedical Engineering and Imaging Sciences, King’s College London, Westminster Bridge Road, London, SE1 7EH UK; 2 Department of Forensic and Neurodevelopmental Sciences, Institute of Psychiatry, Psychology and Neuroscience, King’s College London, London, UK; 3 Oxford Institute of Clinical Psychology Training and Research, Medical Sciences Division, University of Oxford, Oxford, UK; 4 Division of Psychiatry, Department of Brain Sciences, Imperial College London, London, UK; 5 National Female Hormone Clinic, South London and Maudsley National Health Service Foundation Trust, London, UK

**Keywords:** EPDS, Depression, Anxiety, Perinatal, Factor analysis

## Abstract

Timely and accurate detection of perinatal mental health problems is essential for the wellbeing of both mother and child. Growing evidence has suggested that the Edinburgh Postnatal Depression Scale (EPDS) is not a unidimensional measure of perinatal depression, but can be used to screen for anxiety disorders. We aimed to assess the factor structure of the EPDS in 3 different groups of women: *n* = 266 pregnant women at high-risk of depression (“Perinatal Stress Study”), *n* = 471 pregnant women from a community sample, and *n* = 637 early postnatal women from a community sample (“developing Human Connectome Project”). Exploratory factor analysis (40% of each sample) and confirmatory factor analysis (60% of each sample) were performed. The relationship between EPDS scores and history of mental health concerns was investigated. Results suggested that a 3-factor model (depression, anxiety, and anhedonia) is the most appropriate across groups. The anxiety subscale (EPDS-3A) emerged consistently and was related to maternal history of anxiety disorders in the prenatal sample (*W* = 6861, *p* < 0.001). EPDS total score was related to history of mental health problems in both the prenatal (*W* = 12,185, *p* < 0.001) and postnatal samples (*W* = 30,044, *p* < 0.001). In both high-risk and community samples in the perinatal period, the EPDS appears to consist of depression, anxiety, and anhedonia subscales. A better understanding of the multifactorial structure of the EPDS can inform diagnosis and management of women in the prenatal and postnatal period. Further research is required to validate the EPDS-3A as a screening tool for anxiety.

**Supplementary Information:**

The online version contains supplementary material available at 10.1007/s00737-021-01153-0.

## Introduction

Mood and anxiety disorders in the perinatal period affect up to a quarter of women (Howard and Khalifeh 2020). Timely and accurate diagnosis is essential (Kroenke 2020), as early interventions have the greatest potential to improve the wellbeing of mothers and children (Phua et al. 2017; Letourneau et al. 2017). However, as it has been estimated that over half of cases go undetected (National Childbirth Trust 2017; Sudhanthar and Thakur 2019), guidelines have recommended screening and case-finding strategies (National Institute for Health and Care Excellence 2014; Austin et al. 2017; Scottish Intercollegiate Guidelines Network (SIGN), 2012; American College of Obstetricians and Gynecologists 2018).

However, screening strategies to date have focused predominantly on perinatal depression. This is a significant shortcoming, as exposure to prenatal maternal anxiety has detrimental behavioural and cognitive effects on the offspring (van der Zee-van den Berg et al. 2019; O’Donnell et al. 2017) and associated changes in early brain development (Lautarescu et al. 2020). Further, prenatal anxiety is associated with an increased risk for severe postnatal depression (Norhayati et al. 2015). A recommendation to expand antenatal screening to include a tool to assess for anxiety may be impractical in the context of antenatal clinics that are already under significant pressure. An alternative would be to adapt our current screening tools to identify potential anxiety disorders.

The most commonly used screening questionnaire for perinatal depression is the 10-item self-rating Edinburgh Postnatal Depression Scale (EPDS). This was originally developed for postnatal depression (Cox et al. 1987), but its use has since been expanded to prenatal populations (Kozinszky and Dudas 2015; Vázquez and Míguez 2019; Smith-Nielsen et al. 2018). A total score of 13 or more is typically considered to indicate depressive symptoms (Cox et al. 1987; Milgrom and Gemmill, 2015), but a recent meta-analysis suggested that a cut-off of 11 or more maximises combined sensitivity and specificity across reference standards (Levis et al. 2020).

Growing evidence from factor analysis studies suggests that the EPDS is not a unidimensional measure of depression and may be a useful tool for screening for perinatal anxiety (Matthey 2008). More specifically, 3 of the EPDS questions, namely items 3 (“I have blamed myself unnecessarily when things went wrong”), 4 (“I have been anxious or worried for no good reason”) and 5 (“I have felt scared or panicky for no very good reason”), are suggested to constitute an anxiety subscale, called the EPDS-3A. In perinatal women, the EPDS-3A has been found in studies using the English version of the EPDS (Ross et al 2003; Jomeen and Martin 2007; Matthey 2008; Tuohy and McVey 2008; Cunningham et al. 2015) as well as studies using translated versions of the EPDS such the Chinese (Lau et al. 2010), Spanish (Hartley et al. 2014), Japanese (Kubota et al. 2014), Hebrew (Bina and Harrington 2016) or Danish (Smith-Nielsen et al. 2018) versions. It is important to note that some studies do not find the EPDS-3A as a separate factor (e.g. Coates et al. 2017; Phillips et al. 2009), some report an anxiety factor including other items (e.g. Adouard et al. 2005) and some report that a one-factor model is the best fit for the data (e.g. Lydsdottir et al. 2019).

A growing number of researchers have suggested a separate analysis of the EPDS-3A score to screen for perinatal anxiety (Jomeen and Martin 2005; Swalm et al. 2010; Matthey 2008; Phillips et al. 2009; Tuohy and McVey 2008). High scores on the EPDS-3A have been associated with anxiety disorders (Matthey 2008), being a “worrier” (Swalm et al. 2010) and are more strongly associated with anxiety than depression scores (Loyal et al. 2020). Further, the EPDS-3A has the potential to be particularly helpful in detecting anxiety disorders without comorbid depression, in women who may otherwise not reach the total cut-off score necessary for further action (Matthey 2008; Muzik et al. 2000). A cut-off of 6 or more was validated in a postnatal community sample (Matthey 2008), while a cut-off of 4 or more was validated in a sample of women with unsettled infants (Phillips et al. 2009). However, some researchers have voiced concerns regarding the suitability of the EPDS-3A as a screening measure (Adhikari et al. 2020; van der Zee-van den Berg et al.^2019^; Matthey and Agostini 2017, Matthey et al. 2013).

Although previous studies have examined the factor structure of the EPDS, the methodology used for exploratory factor analysis (EFA) and confirmatory factor analysis (CFA) has been inconsistent. Most EFA studies have incorrectly used orthogonal rotations, which assume that variables are not correlated (e.g. Swalm et al. 2010; Maroto-Navarro et al. 2005; Mazhari and Nakhaee 2007; Vivilaki et al. 2009; Flom et al. 2018) or principal component analysis, a technique more appropriate for data reduction (e.g. Brouwers et al. 2001; Adouard et al. 2005; Zhong et al. 2014; Agampodi and Agampodi 2013; Töreki et al. 2013; Lau et al. 2010; Montazeri et al. 2007; Chabrol and Teissedre 2004; Matthey 2008). Other studies have used varying methods including polychoric correlation matrices (Lydsdottir et al. 2019), or Pearson correlation matrices with maximum likelihood extraction (MLE, Kubota et al. 2014, 2018; Phillips et al. 2009; Stasik-O’Brien et al. 2019; Coates et al. 2017), ordinary least squares (Chiu et al. 2017) or principal axis factoring (Tuohy and McVey 2008; Pop et al. 1992). CFA studies have used either MLE (Zhong et al. 2014; Flom et al. 2018; Hartley et al. 2014; Kozinsky and Dudas 2015) or weighted least squares methods (Jomeen and Martin 2007; Kwan et al. 2015; Lydsdottir et al. 2019; King 2012; Gutierrez-Zotes et al. 2018; Martin and Redshaw 2018).

The variability in methodology may explain differences in results reported in the literature.

Other methodological limitations in previous studies include small sample sizes, treating the EPDS as an interval rather than ordinal scale, not reporting cross-loadings, not accounting for non-normally distributed data, not reporting the frequency of responses per each individual item, and performing CFA on the same sample on which EFA was done. Further, most studies have focused on postpartum women, with only two UK-based studies investigating the factor structure of the EPDS antenatally (Jomeen and Martin 2005, *n* = 101; Jomeen and Martin 2007, *n* = 148).

In this study, we aim to overcome some of the methodological shortcomings of previous research and assess, both pre- and postnatal, the factor structure of the EPDS in several subsamples, including self-identified high-risk women and community samples. As a past history of anxiety is a risk factor for perinatal anxiety (Leach et al. 2017; Field 2018), a secondary aim of our study was to assess whether the EPDS-3A is associated with maternal history of anxiety disorders.

## Methods

### Participants

Participants were recruited between March 2015 and March 2020 as part of the developing Human Connectome Project (dHCP, community sample) and the Perinatal Stress Study (high-risk sample). Ethical approval was obtained from the Riverside NHS Research Ethics Committee in the UK (14/LO/1169 and 18/LO/0786).

The dHCP is a large-scale neuroimaging project, with eligibility criteria including pregnant women (aged 16 years or older), with a gestational age of 20–42 weeks, and newborn infants aged 24–44 weeks. The Perinatal Stress Study included pregnant women (any trimester) who self-identified as experiencing low mood during pregnancy (Supplement).

### Measures

All participants were asked to complete the English version of the EPDS. Total scores were calculated with cut-offs set at 11 or more and at 13 or more. EPDS-3A scores were calculated with cut-offs set at 4 or more and 6 or more.

For the Perinatal Stress Study, women completed the EPDS online, alongside a short demographics questionnaire (maternal age, gestational age, and GP details). Eligible pregnant women (total EPDS 13 or more, BMI < 30, no contraventions for MRI) were invited to take part in the dHCP (Supplement).

For the dHCP sample, women were asked to complete the EPDS at the time of their visit to St Thomas’ Hospital in London for an MRI scan (prenatally and/or postnatally). Participants also completed a questionnaire pack which included demographics, medical history, and mental health history. Participant history of mental health concerns (coded as binary yes/no) was determined based on a combination of multiple sources: maternal self-report (in the questionnaire pack), maternity notes, and mental health records from South London and Maudsley NHS Foundation Trust (Supplement). There are no measures of current depression or anxiety symptomatology other than the EPDS in this study.

### Statistical analysis

Data analysis was performed using R (R core team 2018). Throughout the manuscript, “ < ” is used to signify “less than” and “ > ” is used to signify “greater than.” Each dataset was divided randomly into two subsets: 40% for EFA and 60% for CFA. Listwise deletion was used for handling missing data, and only questionnaires with full data were included in the analysis (McNeish 2017). To evaluate the internal consistency of the instrument, Cronbach’s alpha (α) was calculated (acceptable value above 0.70). We also calculated McDonald’s omega (ω) (Hayes and Coutts 2020), as it has been suggested that α is only informative in restrictive settings (Raykov 2004). Raincloud plots were used for visualisation (Allen et al. 2019). Raincloud plots combine split-half violins (showing the probability density of the data), boxplots (showing the median and interquartile range), and raw data points jittered for improved visibility.

To ensure data were suitable for factor analysis, we calculated the Kaiser–Meyer–Olkin measure of sampling adequacy (acceptable limit of > 0.5) and the Bartlett test of sphericity (*p* < 0.05 indicating that the correlations between items are sufficiently large). To detect multicollinearity, we calculated the determinant of the correlation matrix (acceptable value above 0.00001) (Watkins 2018; Jackson et al. 2009; Dziuban and Shirkey 1974).

In subsamples where the frequency of positive responses on item 10 (self-harm) was small, factor analysis was performed on only 9 items, to avoid calculation of potentially negative eigenvalues that would yield non-positive definite matrices (Wothke 1993, as per Chiu et al. 2017; Flom et al. 2018).

#### EFA

EFA was conducted on 40% of the sample. The number of factors for EFA was determined using the conventional Kaiser criteria (eigenvalues above 1), a scree plot, and parallel analysis using Minimum Rank Factor Analysis (MRFA, Shapiro and Berge 2002). MRFA was chosen due to its putative superiority in the identification of the number of factors for ordinal data and performance relative to methods such as Horn’s parallel analysis or those based on principal axis factoring (Baglin 2014). As the variables are ordinal, we used polychoric correlations to correct for bias (Holgado-Tello et al. 2010). However, given that the majority of analyses reported to date have treated the EPDS as an interval scale, we also repeated our analysis using a Pearson correlation matrix (Supplement).

The EFA was conducted using MLE with non-orthogonal oblique (oblimin) rotation, as the variables are expected to be correlated (Jomeen and Martin 2005). All loadings of 0.3 or more (including cross-loadings) were included. We applied a cut-off of 0.3 (Howard 2016; Martin and Thompson 1999, 2000; Martin et al. 2004) to generate a more complete psychological interpretation of data (Jomeen and Martin 2005), while a coefficient of 0.5 or more was used to indicate substantial factor loadings. A factor solution was considered meaningful if it explained at least 50% of variance (Streiner 1994).

An additional EFA was conducted on all 10 items using the whole sample (*n* = 1190), to determine whether there was a common factor structure for women across the perinatal period. This included all high-risk participants and one timepoint (randomly selected) for each participant in the community sample.

#### CFA

CFA was conducted on the remaining 60% of the data using weighted least squares mean and variance (WLSMV), which uses polychoric correlations and robust corrections to account for ordinal and non-normally distributed data (e.g. Lydsdottir et al. 2019; Albuquerque et al. 2017; Martin and Redshaw 2018). As much of the previous literature has used MLE, we also performed CFA using this method (results reported in Supplement).

To test the model fit, we used chi-square statistics, the comparative fit index (CFI, Bentler 1990), and Tucker-Lewis index (TLI, Tucker and Lewis 1973), with values above 0.95 indicating a good fit (Hu and Bentler 1999) and the Root Mean Square Error of Approximation (RMSEA, Schumacker and Lomax 2010), with values under 0.05 indicating adequate fit (Schumacker and Lomax 2010). Goodness of fit was also considered based on the clearest factor structure (i.e. items loading highly on only one factor, and few cross-loadings), plausibility, and interpretability.

In addition to the models suggested by the EFA, a number of models chosen to reflect the wide variety of solutions from the literature were also examined using CFA: 1-factor model (Cox et al. 1987; Lydsdottir et al. 2019); bifactorial model containing depression and anxiety (Phillips et al. 2009; Matthey 2008); bifactorial model containing depression and anhedonia (Zhong et al. 2014); a 3-factor model containing depression, anxiety, and self-harm (Brouwers et al. 2001); and three 3-factor models containing anhedonia, anxiety, and depression (Lau et al. 2010; Kubota et al. 2014; Tuohy and McVey 2008).

#### Maternal history of mental health concerns

To account for ordinal non-normally distributed data, the relationship between EPDS scores and history of mental health concerns was assessed using Wilcoxon rank sum tests, and effect sizes were calculated using Vargha and Delaney’s A (vd.a, Vargha and Delaney 2000, see Supplement). As the EPDS only asks about symptoms over the last 7 days, for longitudinal cases (i.e. where more than one EPDS was completed prenatally or postnatally), the highest score was selected for this analysis (see Supplement).

## Results

### Descriptive statistics

In total, 1374 EPDS questionnaires (Table 1) were available for factor analysis (*n* = 266 high-risk sample, *n* = 1108 community sample). EPDS total scores in the community sample were lower both prenatally (5.10 ± 4.33; 3.89 ± 4.11) and postnatally (5.56 ± 4.38, 6.54 ± 3.78) than in the high-risk prenatal sample (15.52 ± 5.25). The distribution of scores for item 10 (“The thought of harming myself has occurred to me”) was markedly different between the groups, with the community sample answering “Never” in 96.17% and 96.86% of the cases (prenatal and postnatal), while this was the case for only 66.16% of the high-risk sample (Table S2). In the community postnatal sample, the EPDS total score was higher in mothers of babies born extremely preterm (i.e. under 28 weeks), with a mean EPDS total score of 10.13 ± 5.36, than in mothers of babies born at term (i.e. over 37 weeks), with a mean EPDS total score of 5.22 ± 4.12 (see Supplement). As per the methodology described above, the factor analysis was performed on 10 items in the high-risk sample, and 9 items in the community sample.

**Table 1:** Descriptive statistics

	High-risk (Perinatal Stress)	Community (dHCP)			
Measures	Prenatal online	Prenatal Visit 1	Prenatal Visit 2	Postnatal Visit 1	Postnatal Visit 2
N	266	473*	28	640*	24
EPDS total score					
Mean (SD)	15.52 (5.25)	5.10 (4.33)	3.89 (4.11)	5.56 (4.38)	6.54 (3.78)
Range	0–28	0–24	0–16	0–28	1–16
n 11 or more	223	56	2	75	3
n 13 or more	191	32	2	48	1
EPDS-3A score					
Mean(SD)	6.17 (1.91)	2.72 (1.97)	1.93 (1.84)	2.71 (1.98)	3.43 (1.97)
Range	0–9	0–9	0–6	0–8	0–7
n 4 or more	246	151	4	210	12
n 6 or more	176	42	2	64	4
Maternal age at enrolment (years)					
Mean (SD)	30.52 (5.67)	33.79 (4.22)	32.75 (4.52)	33.81 (4.82)	32.96 (4.53)
Range	18–43	20–46	23–39	17–52	23–41
n adolescent mothers (< 20 years)	7	0	0	3	0
Missing values (*n*)	13	0	0	2	0
Gestational age at EPDS (w)					
Mean (SD)	21.72(8.06)	29.65 (4.22)	32.51 (3.34)	40.00 (3.64)	41.57 (1.42)
Range	4–40	20.86–40.29	26.71–37.43	26.71–45.14	38.43–44.00
Missing values (*n*)	7	11	2	6	1
Maternal ethnicity	N/A				
White British		231	14	261	10
White Other		132	10	155	4
Black/Black British		17	0	93	8
Asian/Asian British		49	3	67	0
Mixed ethnic group		16	1	24	1
Other		10	0	26	1
Missing values (*n*)		18	0	14	0
History of MH	N/A	148	12	167	5
Ever been treated for MH	N/A	90	7	86	2
Ever been under psych^	N/A	22	3	26	0
Ever hospitalised for MH^	N/A	3	0	4	0
Self-reported ^	N/A	48	4	53	2
History of depression	N/A	80	6	104	3
History of anxiety	N/A	58	5	51	0
Maternal age at leaving formal education	N/A				
Mean (SD)		23.3 (3.81)	24.88 (4.99)	23.24 (4.51)	23.58 (4.88)
Missing values (*n*)		13	3	34	5
Maternal BMI at enrolment	N/A				
Mean (SD)		22.68 (2.89)	22.3 (2.54)	24.38 (4.62)	26.25 (5.77)
Missing values (*n*)		17	0	84	1
Maternal smoking (*n*)	N/A				
No		432	28	568	21
No, stopped		18	0	31	3
Yes		5	0	16	0
Missing values		18	0	25	0
Maternal alcohol consumption (*n*)	N/A				
No		384	24	542	2
Yes		63	4	54	21
Missing values		26	0	44	1

*w* weeks, *MH* (poor) mental health, *psych* psychiatric services, *BMI* body mass index, *SD* standard deviation; self-reported = do you have a history of ADHD, bipolar disorder, depression, autism, or schizophrenia; smoking and drinking were measured using the questions “Does the mother currently smoke/drink alcohol”.

*Data for all individual questions were available for *n* = 471 (prenatal visit 1) and *n* = 637 (postnatal visit 1), which is the data included in the factor analysis after listwise deletion).

^questions were only asked if answer to “Ever been treated…” was yes.

### Factor analysis

#### Reliability

The Cronbach’s α internal reliability coefficients for the EPDS were good for (a) the 10-item questionnaire for the prenatal high-risk sample 0.85 (95% CI 0.82–0.87), (b) the 9-item questionnaire for the prenatal community sample 0.87 (95% CI 0.85–0.89) and (c) the postnatal community sample 0.85 (95% CI 0.83–0.87). Values were similar for McDonald’s ω (Supplement).

#### EFA

EFA was conducted on 40% of each sample (*n* = 106 prenatal high-risk, *n* = 188 prenatal community, *n* = 255 postnatal community). All criteria for factor analysis were met (Table S3). Both 2- and 3-factor models were examined for all samples (Table 2).

**Table 2:** EFA using polychoric correlation matrices

	Prenatal high-risk					Prenatal community					Postnatal community				
	2-factor		3-factor			2-factor		3-factor			2-factor		3-factor		
	D	AH	D	AX	AH	D	AH	D	AX	AH	D	AX	D	AX	AH
Variance accounted for by model	51%		56%			63%		61%			57%		58%		
Variance accounted for by each factor	35%	16%	25%	16%	15%	27%	32%	26%	18%	16%	34%	23%	18%	23%	17%
1 Able to laugh		**0.53**			**0.55**		**0.87**			**0.96**	**0.93**				**0.74**
2 Look forward		**1.00**			**0.95**		**1.04**	**0.61**		**0.59**	**0.78**				**0.88**
3 Blame self	**0.54**			**0.74**		**0.64**			**0.61**			**0.62**		**0.59**	
4 Anxious/worried	0.35			**0.76**		**0.93**			**0.85**			**0.81**		**0.85**	
5 Scared/panicky	**0.54**			**0.58**		**0.59**			0.45			**0.84**		**0.85**	
6 Overwhelmed	**0.67**		**0.51**			0.48	0.36		0.40	0.32	0.46	0.40		0.46	0.36
7 Difficulty sleep	**0**.**83**		**0.67**			0.35	**0.62**	**0.95**			**0.62**		0.45		
8 Sad or miserable	**0.68**	0.33	**0.69**		0.36	0.33	**0**.**59**	**0.84**			**0.72**		**0.60**		
9 Unhappy/crying	**0.74**		**0.83**			**0.59**		**0.54**	0.38		**0.67**		**0.98**		
10 Self-harm	**0.80**		**0.75**			–-	––	––	––	––	––	––	––	––	––

*D* depression, *AH* anhedonia, *AX* anxiety.

*substantial factor loadings (0.5 or more) in bold.

For full list of questions, see Supplement.

In all 3 samples, the 3-factor EFA revealed distinct factors for anhedonia, anxiety, and depression (Table 2), while the 2-factor structure differed between the groups (i.e. anhedonia and depression in the high-risk sample, anxiety and depression in the postnatal community sample, and no clear solution in the prenatal community sample). All factors were positively correlated in all samples (Supplement). The models were similar for the EFA performed with a Pearson correlation matrix (Table S4). The EFA conducted on the whole sample (*n* = 1190) revealed a 2-factor solution including depression (items 1, 2, 6, 7, 8, 9) and anxiety (items 3, 4, 5) and a 3-factor solution including anhedonia (items 1, 2), anxiety (items 3, 4, 5) and depression (items 8, 9) (Table S6).

#### CFA

CFA was conducted on 60% of the sample (*n* = 160 prenatal high-risk, *n* = 283 prenatal community, *n* = 382 postnatal community). Across all groups, the model with the poorest fit was the unifactorial model (chi-square *p* values < 0.001, smallest CFI and TLI values, RMSEA poor fit, largest SRMSR values), followed by Zhong et al. (2014) 2-factor model of anhedonia and depression (Table 3). This was supported by the results of the MLE analysis (Table S5).

**Table 3:** CFA using WLSMV

Model tested	Sample	Fit indices						
		*X*^2^	P*	df	CFI	TLI	RMSEA (LO90, HI90)	SRMSR
EFA 3 factors								
AH (1,2), AX (3,4,5), D (7–10)	High risk A	36.87	0.045	24	0.986	0.979	0.058 (0.009–0.094)	0.053
AH (1), AX (3,4,5), D (7–8)	Community A	^	^	^	^	^	^	^
AH (1,2), AX (3,4,5), D (8,9)	Community P	20.73	0.036	11	0.995	0.991	0.048 (0.012–0.080)	0.032
EFA 2 factors								
AH (1,2), D(3, 5–10)	High risk A	39.59	0.043	26	0.984	0.978	0.057 (0.011-0.092)	0.054
AH (1,2,7,8), D (3,4,5,9)	Community A	63.21	< 0.001	19	0.968	0.953	0.091 (0.067–0.116)	.0.059
D (1,2, 7–9), AX (3,4,5)	Community P	61.93	< 0.001	19	0.982	0.973	0.077 (0.056–0.099)	0.049
One factor	High risk A	141.94	< 0.001	45	0.897	0.867	0.139 (0.115–0.163)	0.093
	Community A	75.17	< 0.001	27	0.987	0.983	0.080 (0.059–0.101)	0.062
	Community P	198.5	< 0.001	27	0.936	0.915	0.129 (0.113–0.146)	0.077
Phillips 2 factors								
D (1,2,6–10), AX (3,4,5), same as EFA	High risk A	-	-	-	-	-	-	-
D (1,2,6–9), AX (3,4,5)	Community A	42.56	0.021	26	0.996	0.994	0.048 (0.018–0.072)	0.045
D (1,2, 6–9), AX (3,4,5)	Community P	82.58	< 0.001	26	0.975	0.966	0.076 (0.058–0.094)	0.049
Zhong 2 factors								
AH (1,2), D (3–10)	High risk A	121.66	< 0.001	34	0.915	0.888	0.127 (0.103–0.152)	0.085
AH (1,2), D (3–9)	Community A	69.06	0.001	26	0.989	0.984	0.077 (0.055–0.099)	0.057
AH (1,2), D (3–9)	Community P	160.6	< 0.001	26	0.942	0.919	0.117 (0.100–0.134)	0.066
Brouwers 2–3 factors								
D (1,2,8), AX (3,4,5), SH (10)	High risk A	^	^	^	^	^	^	^
D (1,2,8), AX (3,4,5)	Community A	12.43	0.133	8	0.997	0.994	0.044 (0.000–0.090)	0.042
D (1,2,8), AX (3,4,5)	Community P	33.56	< 0.001	8	0.982	0.966	0.092 (0.061–0.125)	0.051
Lau 3 factors								
AH (1,2), AX (3,4,5), D (6–10)	High risk A	51.76	0.015	32	0.981	0.973	0.062 (0.028–0.092)	0.058
AH (1,2), AX (3,4,5), D (6–9)	Community A	36.07	0.054	24	0.997	0.995	0.042 (0.000–0.069)	0.038
AH (1,2), AX (3,4,5), D (6–9)	Community P	41.73	0.014	24	0.992	0.988	0.044 (0.020–0.066)	0.035
Tuohy 3 factors								
AH (1,2),AX (3,4,5),D (7–10), same as EFA	High risk A	-	-	-	-	-	-	-
AH (1,2), AX (3,4,5), D (7–9)	Community A	26.63	0.064	17	0.997	0.995	0.045 (0.000–0.076)	0.039
AH (1,2), AX (3,4,5), D (7–9)	Community P	27.14	0.056	17	0.996	0.993	0.040 (0.000–0.066)	0.033
Kubota 3 factors								
AH (1,2), AX (3,4,5), D (7–9)	High risk A	30.21	0.025	17	0.984	0.974	0.070 (0.025–0.110)	0.050
AH (1,2), AX (3,4,5), D (7–9) Tuohy	Community A	-	-	-	-	-	-	-
AH (1,2), AX (3,4,5), D (7–9) Tuohy	Community P	-	-	-	-	-	-	-

*is sensitive to sample size, and can be significant for large samples, ^ model is not identified, *A* antenatal/prenatal, *P* postnatal, *D* depression, *AX* anxiety, *AH* anhedonia, *SH* self-harm, *SRMSR* Standardised Root Mean Square Residual. < less than.

Across all groups, the model with the best fit was the 3-factor model including anhedonia (items 1, 2), anxiety (items 3, 4, 5), and depression (items 7, 8, 9 and 10 where included in the analysis). This was the 3-factor model obtained through the EFA on the prenatal high-risk sample, as well as Tuohy and McVey’s (2008) and Kubota et al. (2014) models. Lau et al. (2010) 3-factor model was also a good fit (similar to models above, but including item 6 in the depression factor).

### EPDS and history of mental health (community sample)

**Fig. 1:**
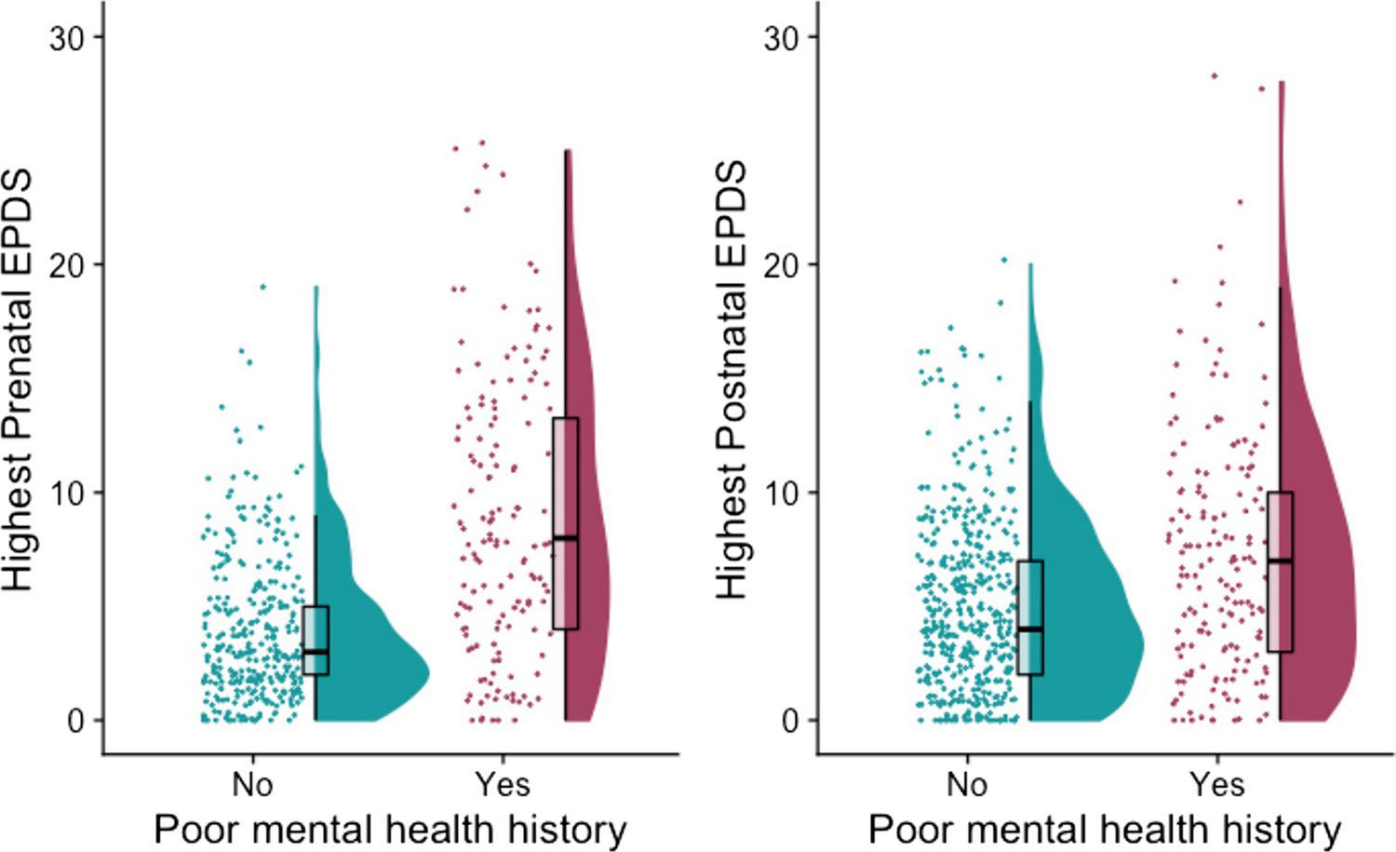
Raincloud plots showing distribution of highest prenatal and postnatal EPDS scores in women with and without a history of mental health conditions. For each group, jittered raw data are shown on the left; boxplots with median and interquartile range are shown in the middle; and density plots are shown on the right

On average, the highest prenatal EPDS total score was higher in those with a history of mental health conditions (*n* = 148, *M* = 8.81, SD = 6.16) than in those without a history *(n* = 325, *M* = 3.88, SD = 3.18), *W* = 12,185, *p* < 0.001, vd.a = 0.253 (large effect size). This was also the case in the postnatal sample, where the highest EPDS total score was higher in those with a history of mental health conditions (*n* = 167, *M* = 7.25, SD = 5.43) than in those without a history (*n* = 473, *M* = 5.03, SD = 3.80), with *W* = 30,044, *p* < 0.001, vd.a = 0.382 (small effect size) (Fig. 1) (Supplement).

### EPDS-3A

The percentage of women with high EPDS-3A scores but EPDS total scores under threshold varied substantially based on the applied cut-offs (Table S7). For example, when using the EPDS-3A cut-off validated in a community sample (i.e. 6 or more, Matthey 2008) and the EPDS total cut-off recommended by a recent meta-analysis (i.e. 11 or more, Levis et al. 2020), the percentage of women that may have anxiety symptoms but not score high enough on the EPDS total to warrant further assessment ranged between 1.90 and 3.38%. However, when using the EPDS-3A cut-off validated in a sample of women with unsettled infants (i.e. 4 or more, Phillips et al. 2009) and the total EPDS original validated cut-off of 13 or more (Cox et al. 1987), these numbers rose to a range of 20.67–26.42%.

**Fig. 2:**
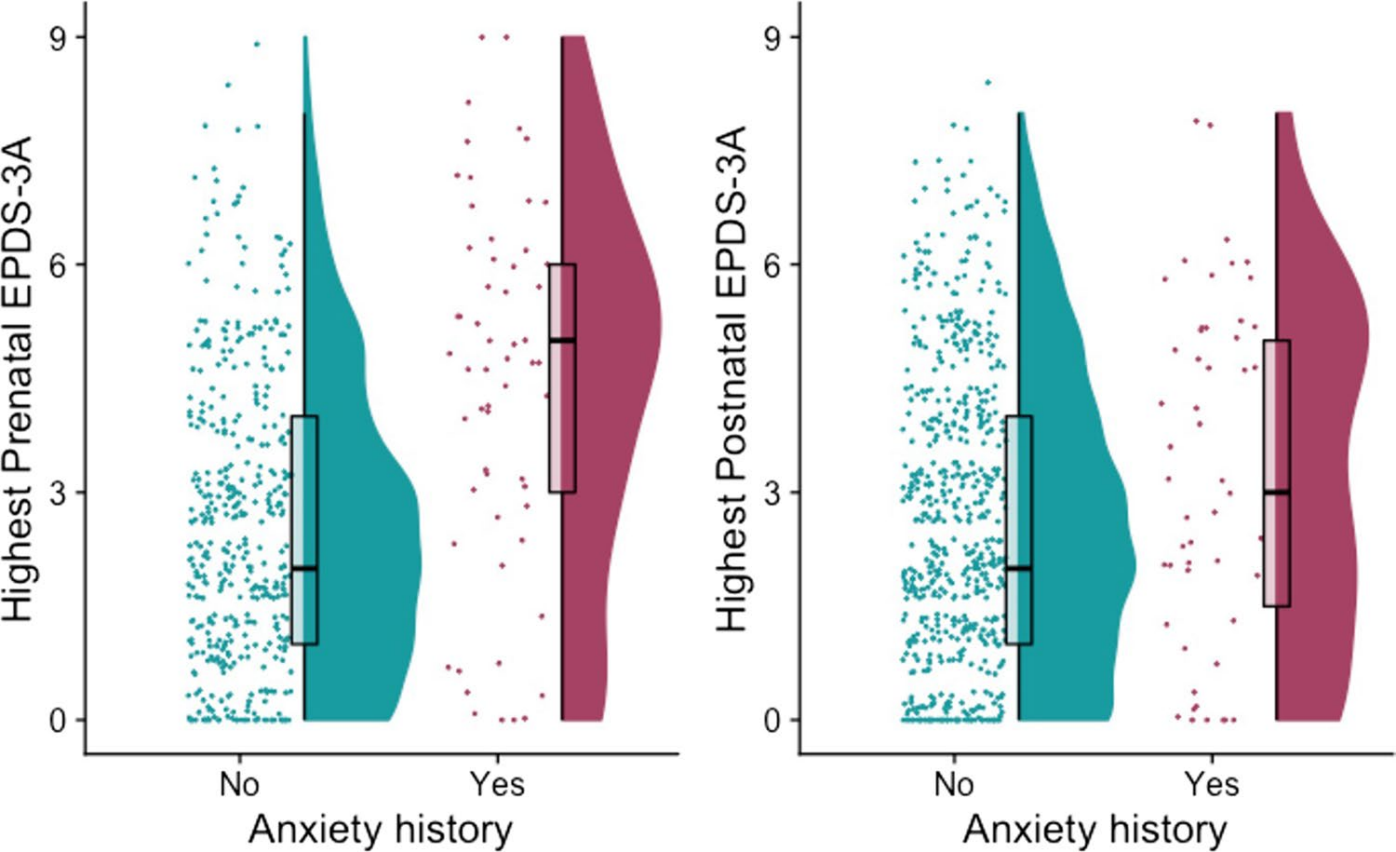
Raincloud plots showing distribution of the highest prenatal and postnatal EPDS-3A scores in women with and without a history of anxiety disorders. For each group, jittered raw data are shown on the left; boxplots with median and interquartile range are shown in the middle; and density plots are shown on the right

On average, the highest prenatal EPDS-3A score was higher in those with a history of anxiety disorders (*n* = 58, *M* = 4.37, SD = 2.47) than in those without a history (*n* = 415, *M* = 2.57, SD = 1.93), *W* = 6861, *p* < 0.001, vd.a = 0.285 (medium effect size). This was also evident in the postnatal sample, with higher EPDS-3A scores in those with a history of anxiety disorders (*n* = 51, *M* = 3.25, SD = 2.29) than those without a history (*n* = 589, *M* = 2.68, SD = 1.95), but the difference was not statistically significant, *W* = 12,856, *p* = 0.087, vd.a = 0.429 (negligible effect size) (Fig. 2) (Supplement).

## Discussion

This study represents an exploration of the 3-factor model of the EPDS as administered prenatally and postnatally, in samples including both community and high-risk populations. We found that the 3-factor structure model of the EPDS (anhedonia, anxiety, and depression) was consistent across populations and was similar to that reported in previous studies (Tuohy and McVey 2008; Kubota et al. 2014; Lau et al. 2010). The EPDS-3A consistently emerged as a separate factor and was associated with a prenatal maternal history of anxiety disorders. These findings are important for several reasons.

Firstly, it has been argued that it is important to examine the utility of screening questionnaires in different populations of women at risk of postnatal depression (Austin et al. 2014). A strength of this study was the ability to examine the factor structure of the EPDS at different timepoints (i.e. prenatally and postnatally) and in different prenatal populations (i.e. high risk and community). It is important to note that the difference in setting (i.e. online versus clinical environment) may influence results, as the relative anonymity offered by the online environment could increase participants’ willingness to more accurately disclose sensitive information (Bowling 2005). While our samples did include mothers of infants born prematurely and a small number of adolescent mothers, further research is required to better understand the factor structure of the EPDS in different (at risk) groups as well as different settings.

Secondly, the current study used both polychoric and Pearson correlation matrices for EFA, in addition to WLS and MLE methods for CFA. This rigorous application of different methodologies increased confidence that the 3-factor model of the EPDS is a genuine construct, rather than the result of idiosyncratic methodological choices.

Thirdly, between 1.9 and 26.4% of the women who had screened positive for anxiety symptoms using the EPDS-3A had scored below threshold when using EPDS total score. This percentage was markedly influenced by the applied cut-offs, and the discrepancies highlight the urgent need for consistent validated cut-offs used across research and clinical settings. Given the inconsistencies associated with the EPDS and EPDS-3A, it may be preferable to use validated measures that screen for a variety of mood and anxiety disorders (e.g. the Matthey Generic Mood Questionnaire, Matthey et al. 2019). However, pending further validation studies, the EPDS-3A may be a useful adjunct to our current screening practice and facilitate patient-provider communication about anxiety symptoms, without further increasing the burden on women. This is of particular importance in contexts where no validated anxiety questionnaire is routinely administered in the perinatal period. It is important to note that the EPDS only asks about the last 7 days. A substantial proportion of high scores on the EPDS will reflect only transient symptoms of depression and/or anxiety (Agostini et al. 2019; Matthey and Ross-Hamid 2012).

Fourthly, our study was strengthened by the availability of data on maternal history of psychiatric disorders. This led to the finding of an association between prenatal EPDS-3A and maternal history of anxiety disorders. It remains unclear why postnatal EPDS-3A was unrelated to history of anxiety but this may be due to the postnatal sample consisting largely of women in the very early postnatal period (neonate postmenstrual age = 40.00 ± 3.64 weeks), when anxiety may be more related to specific experiences during labour (Bell and Andersson 2016, Paul et al. 2013). Further research is required in order to determine whether the EPDS-3A is uniquely associated with a history of anxiety disorders relative to other mental health concerns (Supplement). A major limitation of our study is the lack of validated measures of current anxiety symptoms. We recommend that future research includes comprehensive diagnostic interviews and clinical assessments of mental health and psychiatric history, as well as quantitative screening measures of anxiety.

Finally, it is important to note that although the overall factor structure was relatively stable, the exact factor loadings were influenced by analysis choices. Further studies are required to determine the measurement invariance (Widaman 2010) of this instrument.

We believe that studies may also benefit from analysing the relationship between EPDS-3A (or other screening tools) and physiological correlates of anxiety. For example, we are currently exploring the correlation between EPDS-3A score, maternal heart rate variability and neonatal brain development, using data collected from the wider dHCP project. It is hoped that this will enable us to better clarify the clinical relevance of a prenatal maternal EPDS-3A score, and ultimately to better target interventions with positive effects on early brain development.

## Supplementary Information

ESM 1 (DOCX 1.7 kb)

## Data Availability

We are unable to make the data openly available due to ethical constraints.

## Code availability

Code availability is available in the Supplementary Information.

## References

[CR1] Adhikari K, Patten SB, Williamson T, Patel AB, Premji S, Tough S, …Metcalfe A (2020) Assessment of anxiety during pregnancy: are existing multiple anxiety scales suitable and comparable in measuring anxiety during pregnancy? J Psychosom Obstet Gynecol 1–7 10.1080/0167482X.2020.1725462 10.1080/0167482X.2020.1725462 32056477

[CR2] Adouard F, Glangeaud-Freudenthal NMC, Golse B (2005). Validation of the Edinburgh postnatal depression scale (EPDS) in a sample of women with high-risk pregnancies in France. Arch Women’s Ment Health.

[CR3] Agampodi SB, Agampodi TC (2013). Antenatal depression in Anuradhapura, Sri Lanka and the factor structure of the Sinhalese version of Edinburgh Post Partum Depression Scale among pregnant women. PLoS ONE.

[CR4] Agostini F, Matthey S, Minelli M, Dellabartola S, Bonapace S (2019). Transient vs enduring distress in late pregnancy using the EPDS: a brief longitudinal exploratory study. J Reprod Infant Psychol.

[CR5] Albuquerque MR, Corrêa H, Santos W, Romano-Silva MA, Santos LMP (2017). A proposal for a new Brazilian six-item version of the Edinburgh Postnatal Depression Scale. Trends in Psychiatry and Psychotherapy.

[CR6] Allen M, Poggiali D, Whitaker K, Marshall TR, & Kievit RA (2019) Raincloud plots: a multi-platform tool for robust data visualization. Wellcome Open Research 4. 10.12688/wellcomeopenres.15191.1 10.12688/wellcomeopenres.15191.1 PMC6480976 31069261

[CR7] American College of Obstetricians and Gynecologists (2018). Screening for perinatal depression. ACOG Committee Opinion No. 757. Obstet Gynecol.

[CR8] Austin M-P, Highet N and the Expert Working Group (2017) Mental health care in the perinatal period: australian clinical practice guideline. Melbourne: Centre of Perinatal Excellence.

[CR9] Austin MP, & Marcé Society Position Statement Advisory Committee (2014). Marcé International Society position statement on psychosocial assessment and depression screening in perinatal women. Best Pract Res Clin Obstet Gynaecol.

[CR10] Baglin J (2014). Improving your exploratory factor analysis for ordinal data: a demonstration using FACTOR. Pract Assess Res Eval.

[CR11] Bell AF, Andersson E (2016). The birth experience and women's postnatal depression: a systematic review. Midwifery.

[CR12] Bentler PM (1990). Comparative fit indexes in structural models. Psychol Bull.

[CR13] Bina R, Harrington D (2016). The Edinburgh Postnatal Depression Scale: screening tool for postpartum anxiety as well? Findings from a confirmatory factor analysis of the Hebrew version. Matern Child Health J.

[CR14] Bowling A (2005). Mode of questionnaire administration can have serious effects on data quality. J Public Health.

[CR15] Brouwers EP, van Baar AL, Pop VJ (2001). Does the Edinburgh postnatal depression scale measure anxiety?. J Psychosom Res.

[CR16] Chabrol H, Teissedre F (2004). Relation between Edinburgh Postnatal Depression Scale scores at 2–3 days and 4–6 weeks postpartum. J Reprod Infant Psychol.

[CR17] Chiu YHM, Sheffield PE, Hsu HHL, Goldstein J, Curtin PC, Wright RJ (2017). Subconstructs of the Edinburgh Postnatal Depression Scale in a multi-ethnic inner-city population in the US. Arch Women’s Ment Health.

[CR18] Coates R, Ayers S, de Visser R (2017). Factor structure of the Edinburgh Postnatal Depression Scale in a population-based sample. Psychol Assess.

[CR19] Cox JL, Holden JM, Sagovsky R (1987). Detection of postnatal depression. Development of the 10-item Edinburgh Postnatal Depression Scale. Br J Psychiatry.

[CR20] Cunningham NK, Brown PM, Page AC (2015). Does the Edinburgh Postnatal Depression Scale measure the same constructs across time?. Arch Womens Ment Health.

[CR21] Dziuban CD, Shirkey EC (1974). When is a correlation matrix appropriate for factor analysis?. Some Decision Rules Psychological Bulletin.

[CR22] Field T (2018). Postnatal anxiety prevalence, predictors and effects on development: a narrative review. Infant Behav Dev.

[CR23] Flom JD, Chiu YHM, Tamayo-Ortiz M, Schnaas L, Curtin PC, Wright RJ, …Rosa MJ 2018 Subconstructs of the Edinburgh Postpartum Depression Scale in a postpartum sample in Mexico City J Affect Disord 238 142 146 10.1016/j.jad.2018.05.049. 10.1016/j.jad.2018.05.049 PMC6063785 29879609

[CR24] Gutierrez-Zotes A,Gallardo-Pujol D, Labad J, Martín Santos R, García-Esteve L, Gelabert E, …Canellas F 2018 Factor structure of the Spanish version of the Edinburgh postnatal depression scale Actas Espanolas De Psiquiatria 46 5 174 182 30338774

[CR25] Hartley CM, Barroso N, Rey Y, Pettit JW, Bagner DM (2014). Factor structure and psychometric properties of English and Spanish versions of the Edinburgh Postnatal Depression Scale among Hispanic women in a primary care setting. J Clin Psychol.

[CR26] Hayes AF, & Coutts JJ (2020) Use omega rather than Cronbach’s alpha for estimating reliability. But…. Communication Methods and Measures, 14(1), 1–24. 10.1080/19312458.2020.1718629

[CR27] Holgado-Tello FP, Chacón-Moscoso S, Barbero-García I, Vila-Abad E (2010). Polychoric versus Pearson correlations in exploratory and confirmatory factor analysis of ordinal variables. Qual Quant.

[CR28] Howard MC (2016). A review of exploratory factor analysis decisions and overview of current practices: what we are doing and how can we improve?. Int J Hum Comput Interact.

[CR29] Howard LM, Khalifeh H (2020). Perinatal mental health: a review of progress and challenges. World Psychiatry.

[CR30] Hu LT, Bentler PM (1999). Cutoff criteria for fit indexes in covariance structure analysis: conventional criteria versus new alternatives. Struct Equ Modeling.

[CR31] Jackson DL, Gillaspy JA, Purc-Stephenson R (2009). Reporting practices in confirmatory factor analysis: An overview and some recommendations. Psychol Methods.

[CR32] Jomeen J, Martin CR (2005). Confirmation of an occluded anxiety component within the Edinburgh Postnatal Depression Scale (EPDS) during early pregnancy. J Reprod Infant Psychol.

[CR33] Jomeen J, Martin CR (2007) Replicability and stability of the multidimensional model of the Edinburgh Postnatal Depression Scale in late pregnancy. J Psychiatr Ment Health Nurs 14, 319–324. 10.1111/j.1365-2850.2007.01084.x 10.1111/j.1365-2850.2007.01084.x 17430456

[CR34] King PAL (2012). Replicability of structural models of the Edinburgh Postnatal Depression Scale (EPDS) in a community sample of postpartum African American women with low socioeconomic status. Arch Womens Ment Health.

[CR35] Kozinszky Z, Dudas RB (2015). Validation studies of the Edinburgh Postnatal Depression Scale for the antenatal period. J Affect Disord.

[CR36] Kroenke K (2020). Two birds with one stone: joint screening for perinatal depression and anxiety. J Womens Health.

[CR37] C Kubota T Okada B Aleksic Y Nakamura S Kunimoto M Morikawa …T Morita 2014 Factor structure of the Japanese version of the Edinburgh Postnatal Depression Scale in the postpartum period PLoS ONE 9 8 e103941 10.1371/journal.pone.0103941 10.1371/journal.pone.0103941 PMC4121230 25089523

[CR38] C Kubota T Inada Y Nakamura T Shiino M Ando B Aleksic …M Sato 2018 Stable factor structure of the Edinburgh Postnatal Depression Scale during the whole peripartum period: results from a Japanese prospective cohort study Sci Rep 8 1 17659 10.1038/s41598-018-36101-z 10.1038/s41598-018-36101-z PMC6281669 30518774

[CR39] Kwan R, Bautista D, Choo R, Shirong C, Chee C, Mei Saw S, Chong YS, Kwek K, Meaney MJ, Rush AJ, Chen H (2015). The Edinburgh Postnatal Depression Scale as a measure for antenatal dysphoria. J Reprod Infant Psychol.

[CR40] Lau Y, Wang Y, Yin L, Chan KS, Guo X (2010) Validation of the Mainland Chinese version of the Edinburgh Postnatal Depression Scale in Chengdu mothers. Int J Nurs Stud 47, 1139–1151. 10.1016/j.ijnurstu.2010.02.005 10.1016/j.ijnurstu.2010.02.005 20219196

[CR41] A Lautarescu MC Craig V Glover 2020 Prenatal stress: effects on fetal and child brain development stress and brain health: across the Life Course 17 10.1016/bs.irn.2019.11.002 10.1016/bs.irn.2019.11.002 32204831

[CR42] Leach LS, Poyser C, Fairweather-Schmidt K (2017). Maternal perinatal anxiety: A review of prevalence and correlates. Clin Psychol.

[CR43] Letourneau NL, Dennis CL, Cosic N, Linder J (2017). The effect of perinatal depression treatment for mothers on parenting and child development: a systematic review. Depress Anxiety.

[CR44] B Levis Z Negeri Y Sun A Benedetti BD Thombs 2020 Accuracy of the Edinburgh Postnatal Depression Scale (EPDS) for screening to detect major depression among pregnant and postpartum women: systematic review and meta-analysis of individual participant data BMJ 371 10.1136/bmj.m4022 10.1136/bmj.m4022 PMC7656313 33177069

[CR45] Loyal D, Sutter AL, Rascle N (2020). Screening beyond postpartum depression: Occluded Anxiety Component in the EPDS (EPDS-3A) in French Mothers. Matern Child Health J.

[CR46] Lydsdottir LB, Howard LM, Olafsdottir H, Thome M, Tyrfingsson P, & Sigurdsson JF (2019) The psychometric properties of the Icelandic version of the Edinburgh Postnatal Depression Scale (EPDS) when used prenatal. Midwifery 69, 45–51. 10.1016/j.midw.2018.10.009. 10.1016/j.midw.2018.10.009 30396159

[CR47] Maroto-Navarro G, Garcia-Calvente MM, Fernandez-Parra A (2005). Evaluation of mood in the postpartum period with the Edinburgh Postnatal Depression Scale. Int J Clin Health Psychol.

[CR48] Martin CR, Thompson DR (1999). Utility of the Hospital Anxiety and Depression Scale in patients with end-stage renal disease on continuous ambulatory peritoneal dialysis. Psychol Health Med.

[CR49] Martin CR, Thompson DR (2000). A psychometric evaluation of the Hospital Anxiety and Depression Scale in coronary care patients following acute myocardial infarction. Psychol Health Med.

[CR50] Martin CR, Tweed AE, Metcalfe MS (2004). A psychometric evaluation of the hospital anxiety and depression scale in patients diagnosed with end-stage renal disease. Br J Clin Psychol.

[CR51] Martin CR, Redshaw M (2018). Establishing a coherent and replicable measurement model of the Edinburgh postnatal depression scale. Psychiatry Res.

[CR52] Matthey S (2008). Using the Edinburgh Postnatal Depression Scale to screen for anxiety disorders. Depress Anxiety.

[CR53] Matthey S, Ross-Hamid C (2012). Repeat testing on the Edinburgh Depression Scale and the HADS-A in pregnancy: differentiating between transient and enduring distress. J Affect Disord.

[CR54] Matthey S, Fisher J, Rowe H (2013). Using the Edinburgh Postnatal Depression Scale to screen for anxiety disorders: conceptual and methodological considerations. J Affect Disord.

[CR55] Matthey S, Agostini F (2017). Using the Edinburgh Postnatal Depression Scale for women and men—some cautionary thoughts. Arch Womens Ment Health.

[CR56] Matthey S, Souter K, Valenti B, Ross-Hamid C (2019). Validation of the MGMQ in screening for emotional difficulties in women during pregnancy. J Affect Disord.

[CR57] Mazhari S, Nakhaee N (2007). Validation of the Edinburgh Postnatal Depression Scale in an Iranian sample. Archives of Womens Mental Health.

[CR58] McNeish D (2017). Exploratory factor analysis with small samples and missing data. J Pers Assess.

[CR59] Milgrom J, & Gemmill AW (eds) (2015) Identifying perinatal depression and anxiety: evidence-based practice in screening, psychosocial assessment and management. John Wiley & Sons. 10.1002/9781118509722

[CR60] Montazeri A, Torkan B, Omidvari S (2007). The Edinburgh Postnatal Depression Scale (EPDS): translation and validation study of the Iranian version. BMC Psychiatry.

[CR61] Muzik M, Klier CM, Rosenblum KL, Holzinger A, Umek W, Katschnig H (2000). Are commonly used self-report inventories suitable for screening postpartum depression and anxiety disorders?. Acta Psychiatr Scand.

[CR62] National Institute for Health and Care Excellence (2014) Antenatal and postnatal mental health: clinical management and service guidance. Clinical Guidance [CG192]. 31990493

[CR63] National Childbirth Trust (2017) The hidden half: bringing postnatal mental illness out of hiding. https://www.nct.org.uk/sites/default/files/related_documents/NCT%20The%20Hidden%20Half%20shortform.pdf

[CR64] Norhayati MN, Hazlina NN, Asrenee AR, Emilin WW (2015). Magnitude and risk factors for postpartum symptoms: a literature review. J Affect Disord.

[CR65] O’Donnell KJ, Glover V, Lahti J, Lahti M, Edgar RD, Räikkönen K, & O’Connor TG (2017) Maternal prenatal anxiety and child COMT genotype predict working memory and symptoms of ADHD. PloS one 12(6). 10.1371/journal.pone.0177506 10.1371/journal.pone.0177506 PMC5470664 28614354

[CR66] Paul IM, Downs DS, Schaefer EW, Beiler JS, Weisman CS (2013). Postpartum anxiety and maternal-infant health outcomes. Pediatrics.

[CR67] Phillips J, Charles M, Sharpe L, Matthey S (2009). Validation of the subscales of the Edinburgh Postnatal Depression Scale in a sample of women with unsettled infants. J Affect Disord.

[CR68] Phua DY, Kee MK, Koh DX, Rifkin-Graboi A, Daniels M, Chen H, ... & Meaney MJ (2017) Positive maternal mental health during pregnancy associated with specific forms of adaptive development in early childhood: evidence from a longitudinal study. Development and Psychopathology 29(5), 1573–1587. 10.1017/S0954579417001249 10.1017/S0954579417001249 29162171

[CR69] Pop VJ, Komproe IH, Van Son MJ (1992). Characteristics of the Edinburgh post natal depression scale in The Netherlands. J Affect Disord.

[CR70] R Core Team (2018) R: A language and environment for statistical computing. R Foundation for Statistical Computing, Vienna, Austria. URL https://www.R-project.org/.

[CR71] Raykov T (2004). Behavioral scale reliability and measurement invariance evaluation using latent variable modeling. Behav Ther.

[CR72] Ross LE, Evans SG, Sellers EM, Romach MK (2003). Measurement issues in postpartum depression part 1: anxiety as a feature of postpartum depression. Arch Womens Ment Health.

[CR73] Schumacker RE, Lomax RG (2010) A beginner’s guide to structural equation modeling, 3rd edn. Routledge/Taylor & Francis Group. 10.4324/9780203851319

[CR74] Scottish Intercollegiate Guidelines Network (SIGN) (2012) Management of perinatal mood disorders. Edinburgh: SIGN.

[CR75] Shapiro A, Ten Berge JM (2002). Statistical inference of minimum rank factor analysis. Psychometrika.

[CR76] Smith-Nielsen J, Matthey S, Lange T, Væver MS (2018). Validation of the Edinburgh Postnatal Depression Scale against both DSM-5 and ICD-10 diagnostic criteria for depression. BMC Psychiatry.

[CR77] Stasik-O’Brien SM, McCabe-Beane JE, Segre LS (2019). Using the EPDS to identify anxiety in mothers of infants on the neonatal intensive care unit. Clin Nurs Res.

[CR78] Streiner DL (1994). Figuring out factors: the use and misuse of factor analysis. Can J Psychiatry.

[CR79] Sudhanthar S, Thakur K (2019). Postpartum depression screening: are we doing a competent job?. BMJ Open Quality.

[CR80] Swalm D, Brooks J, Doherty D, Nathan E, Jacques A (2010). Using the Edinburgh Postnatal Depression Scale to screen for perinatal anxiety. Arch Womens Ment Health.

[CR81] Töreki A, Andó B, Keresztúri A, Sikovanyecz J, Dudas RB, Janka Z, Kozinszky Z, Pál A (2013). The Edinburgh Postnatal Depression Scale: translation and antepartum validation for a Hungarian sample. Midwifery.

[CR82] Tucker LR, Lewis C (1973). A reliability coefficient for maximum likelihood factor analysis. Psychometrika.

[CR83] Tuohy A, McVey C (2008). Subscales measuring symptoms of non-specific depression, anhedonia, and anxiety in the Edinburgh Postnatal Depression Scale. Br J Clin Psychol.

[CR84] van der Zee-van den Berg AI, Boere-Boonekamp MM, Groothuis-Oudshoorn CG, & Reijneveld SA (2019) The Edinburgh Postpartum Depression Scale: stable structure but subscale of limited value to detect anxiety. PloS one 14(9), e0221894. 10.1371/journal.pone.0221894 10.1371/journal.pone.0221894 PMC6733480 31498818

[CR85] Vargha A, Delaney HD (2000). A critique and improvement of the CL common language effect size statistics of McGraw and Wong. Journal of Educational and Behavioral Statistics.

[CR86] Vázquez MB, Míguez MC (2019). Validation of the Edinburgh postnatal depression scale as a screening tool for depression in Spanish pregnant women. J Affect Disord.

[CR87] Vivilaki VG, Dafermos V, Kogevinas M, Bitsios P, Lionis C (2009) The Edinburgh Postnatal Depression Scale: translation and validation for a Greek sample. BMC Public Health 9, 329. 10.1186/1471-2458-9-329 10.1186/1471-2458-9-329 PMC2748079 19740443

[CR88] Watkins MW (2018). Exploratory factor analysis: a guide to best practice. J Black Psychol.

[CR89] Widaman KF, Ferrer d E, & Conger RD,  (2010). Factorial invariance within longitudinal structural equation models: Measuring the same construct across time. Child Development Perspectives.

[CR90] Wothke W, Bollen KA, Long JS (1993). Nonpositive definite matrices in structural modeling. Testing structural equation models.

[CR91] Q Zhong B Gelaye M Rondon SE Sánchez PJ García E Sánchez …MA Williams 2014 Comparative performance of patient health questionnaire-9 and Edinburgh Postnatal Depression Scale for screening antepartum depression J Affect Disord 162 1 7 10.1016/j.jad.2014.03.028 10.1016/j.jad.2014.03.028 PMC4040145 24766996

