# Supplementary material for: The factor structure of the Edinburgh Postnatal Depression Scale among perinatal high-risk and community samples in London
Source: Arch Womens Ment Health. 2021 Jul 10;25(1):157–69. doi: 10.1007/s00737-021-01153-0 (PMC8784492; doi:10.1007/s00737-021-01153-0)
Supplement: Supplementary file 1 — (DOCX 1.7 kb) [file 737_2021_1153_MOESM1_ESM.docx]

**SUPPLEMENT**

**Title:** The factor structure of the Edinburgh Postnatal Depression Scale among perinatal high-risk and community samples in London

**Journal:** Archives of Women’s Mental Health

**Author names and affiliations:**

Alexandra Lautarescu ^1,2^, Suresh Victor^1^, Alex Lau-Zhu ^3,4^, Serena J. Counsell^1^, A. David Edwards^1^ , Michael C. Craig^2,5^

1 - Department of Perinatal Imaging and Health, Centre for the Developing Brain, School of Biomedical Engineering and Imaging Sciences, King’s College London, London, United Kingdom

2 - Department of Forensic and Neurodevelopmental Sciences, Institute of Psychiatry, Psychology and Neuroscience, King’s College London, London, United Kingdom

3 - Oxford Institute of Clinical Psychology Training and Research, Medical Sciences Division, University of Oxford, Oxford, United Kingdom

4 – Division of Psychiatry, Department of Brain Sciences, Imperial College London, London, United Kingdom

5 - National Female Hormone Clinic, South London and Maudsley National Health Service Foundation Trust, London, United Kingdom

The analysis code is openly available at https://osf.io/hakzp/

1. **Individual items from the EPDS**

Table S1. Individual items from the EPDS

| Item number | Question |
| --- | --- |
| 1 | Been able to laugh and see the funny side of things |
| 2 | Looked forward with enjoyment to things |
| 3* | Blamed myself unnecessarily when things went wrong |
| 4 | Been anxious or worried for no good reason |
| 5* | Felt scared or panicky for no very good reason |
| 6* | Things have been getting on top of me |
| 7* | Been so unhappy that I have had difficulty sleeping |
| 8* | Felt sad or miserable |
| 9* | Been so unhappy that I have been crying |
| 10* | Thought of harming myself has occurred to me |

*reverse scored

1. **Additional information about the study groups**

2.1. Perinatal Stress Study

The main aim of the Perinatal Stress study was a targeted recruitment of women with depressive symptoms. Participants in the Perinatal Stress study were recruited via a flyer directed at pregnant women which stated “If you are (..) experiencing low mood, we would like to invite you to be part of our study”. They completed a very brief online questionnaire, which included the EPDS, as well as brief questions covering maternal age, gestational age, and GP details (no other data was collected for this sample). All pregnant participants who completed the online questionnaire were included in this study. Participants in the Perinatal Stress Study were informed that a member of the research team will contact them to discuss the results and consented to their GP being informed of potential high scores. The EPDS questionnaire was scored by the researcher (AL) following appropriate scoring guidelines. After completing the online questionnaire, all women were contacted by the researcher to be provided with their score, and eligible pregnant women (EPDS 13 or more, BMI<30, no contraventions for MRI) were invited to participate in the dHCP and take part in the fetal and/or neonatal MRI scans. N=29 pregnant women took part in the fetal timepoint of the dHCP and n=5 postpartum women took part in the neonatal timepoint of the dHCP.

2.2. Developing Human Connectome Project

The main aim of the developing Human Connectome Project is to assess brain development in fetuses and neonates. Participants in the dHCP were recruited in obstetric ultrasound clinics, antenatal clinics, postnatal wards, neonatal wards, and through referrals from their medical advisors. Inclusion criteria are noted in the manuscript. Exclusion criteria include: contraindications to MR imaging, babies being too unwell to tolerate a scan, and language difficulties preventing informed consent. Participants in the dHCP completed the EPDS as part of an extensive mother’s questionnaire. Participants completed the EPDS independently in a private room, with no interference from the researcher. Participants were informed that a member of the research team will discuss the results with them and consented to their GP being informed of potential high scores. The EPDS questionnaire was scored by a member of the dHCP research team. Participants were included in the current study if they had completed the EPDS questionnaire at a minimum of one timepoint. Several participants had longitudinal data on EPDS scores (n=225). Most longitudinal participants completed EPDS scores at 2 timepoints (n=38 at two prenatal timepoints, n=23 at two postnatal timepoints, n=148 at one prenatal and one postnatal timepoint), although some participants had EPDS scores at 3 timepoints (n=15 at two prenatal timepoints and one postnatal timepoint, n=1 at three fetal timepoints). Figure S1 presents a summary of the trajectory of scores for descriptive purposes, but further analysis of this is beyond the scope of this paper.

Figure S1. Trajectory of longitudinal EPDS scores for the Perinatal Stress Study (PSS) and dHCP first prenatal timepoint (F), second prenatal timepoint (F2), first postnatal timepoint (N) and second neonatal timepoint (N2). Based on a cut-off of 11 or more, participants scored consistently high (n=23), consistently low (n=163), had decreased scores (i.e. High_Low, n=24), had increased scores (i.e. Low_High, n=13), or a low – high – low pattern (n=2)


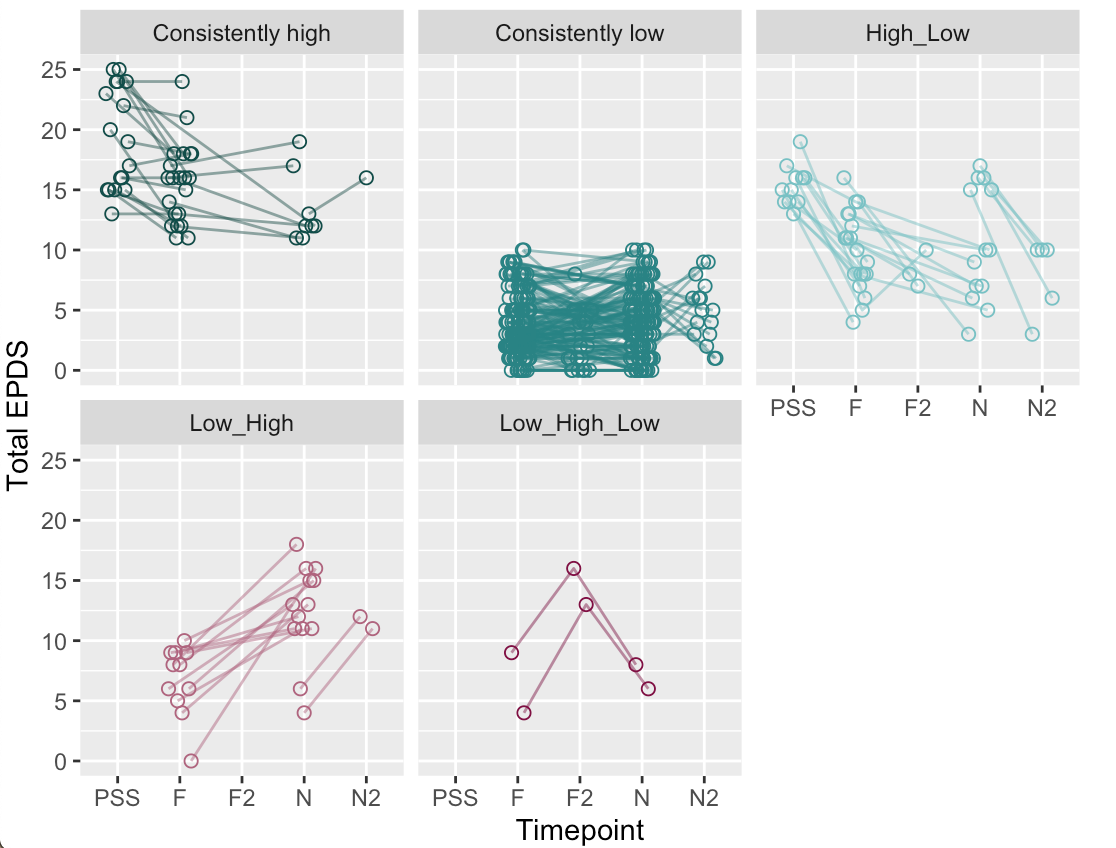


For the group analysis (i.e. sections 3.3. and 3.4. in the manuscript), in cases where longitudinal data was available, we selected the highest prenatal EPDS score and the highest postnatal EPDS scores. In the postnatal sample, the highest total EPDS was almost always from the first postnatal timepoint (with the exception of n=10 where total EPDS was higher at the second neonatal timepoint). In the prenatal sample, the highest total EPDS score was most commonly extracted from the first dHCP prenatal timepoint (with the exception of n=25 where total EPDS was higher at the online PSS questionnaire, n=7 where total EPDS was higher at the second dHCP visit, and n=1 where total EPDS was higher at the third dHCP prenatal visit).

Apart from the EPDS, the dHCP questionnaire pack included demographics, history of medical conditions, and questions regarding history of mental health conditions. More specifically, participants were asked whether they have ever been treated for a mental health condition (yes/no answer) and for those who answered yes, 3 additional questions were asked. These were whether the mother has ever been under psychiatric care, ever been hospitalized for a mental health condition, and whether they have a history of any of the following: ADHD, bipolar disorder, depression, autism, or schizophrenia. To obtain a more comprehensive picture of maternal history of mental health, information was collated from multiple sources including self-report, maternal electronic and hand-held notes, and records from the South London and Maudsley NHS Foundation Trust. In the current study, participant history of anxiety was coded yes/no, with “yes” indicating one or more mentions of anxiety disorder in any of the above-mentioned sources. Participant history of mental health conditions was coded yes/no, with “yes” indicating one or more mentions of *any* mental health condition, including anxiety, depression or positive answers to the maternal questionnaire described above (i.e. ever been treated for mental health, ever been hospitalized for mental health etc.).

As mental health data were partly collected from SLaM records, we were interested in the distribution of participants based on location. The sample included participants from all London boroughs with the exception of City of London, as well as adjacent areas (Figure S2). For the dHCP sample, 62.5% of neonatal participants and 55.5% of fetal participants are from boroughs in which SLaM is a provider of primary and secondary mental health services (i.e. Southwark, Lambeth, Lewisham, Croydon), and 15.3% and 12.5% respectively from boroughs in which SLaM provides other mental health services (i.e. Wandsworth, Bromley, Bexley, Greenwich). Additionally, SLaM also provides national specialist services accepting referrals from anywhere in the country.

Figure S2. Distribution of participants in the perinatal stress study (A), prenatal dHCP (B), postnatal dHCP (C) in London boroughs and surrounding areas.
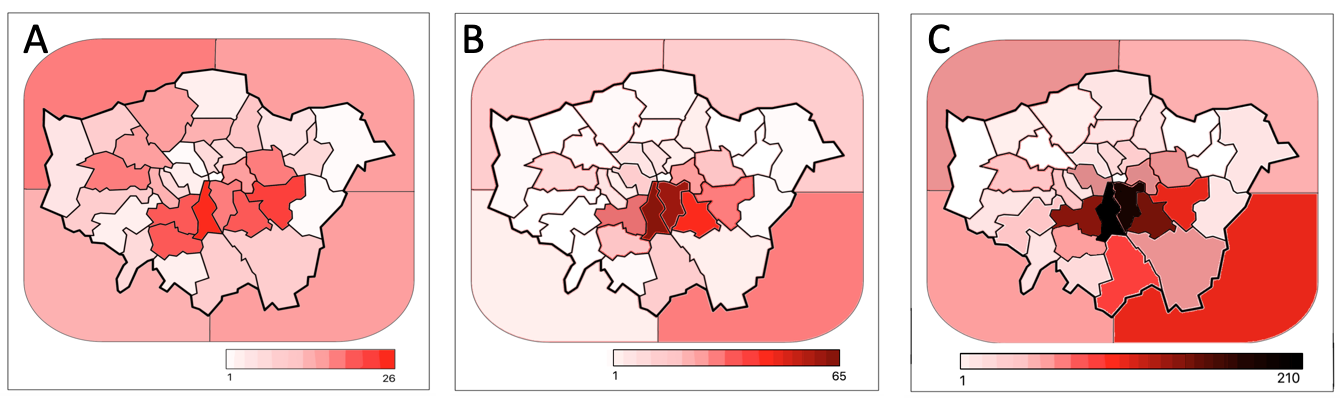


1. **Frequency of responses per each individual item of the EPDS**

We considered it was essential to report the frequency of responses per each individual EPDS item, as recent studies have highlighted the need for this information (Martin 2018) in order to better understand the distributional characteristics of EPDS items

| Table S2. Frequency of responses per each item | | | | |
| --- | --- | --- | --- | --- |
| Question | Response | High risk antenatal | Community antenatal | Community  postnatal |
| 1.“Able to laugh” | 1. As much as always 2. Not quite so much now 3. Definitely not so much 4. Not at all | 26.69  50.37  21.05  1.87 | 89.80  8.91  1.27  0 | 81.16  15.38  2.66  0.78 |
| 2. “Looked forward” | 1. As much as ever 2. Rather less .. 3. Definitely less .. 4. Hardly at all | 16.91  44.73  30.07  8.27 | 87.47  10.19  2.12  0.21 | 84.30  12.87  1.88  0.94 |
| 3. “Blamed self unnecessarily”* | 1. No, never 2. Not very often 3. Yes, some of the time 4. Yes, most of the time | 4.51  20.67  46.24  28.57 | 23.77  49.04  24.62  2.54 | 26.37  43.32  27.94  2.35 |
| 4 “Anxious or worried” | 1. Not at all 2. Hardly ever 3. Yes, sometimes 4. Yes, very often | 3.00  7.51  48.12  41.35 | 33.12  32.05  32.69  2.12 | 35.32  32.49  28.57  3.61 |
| 5 “Scared or panicky”* | 1. No, not at all 2. No, not much 3. Yes, sometimes 4. Yes, quite a lot | 7.51  20.67  46.24  25.56 | 56.05  28.02  13.80  2.12 | 53.84  29.51  15.22  1.41 |
| 6 “Things getting on top of me”* | 1. No, I have been coping 2. No, most of the time.. 3. Yes, sometimes .. 4. Yes, most of the time.. | 4.13  15.78  64.66  15.41 | 37.36  44.79  17.19  0.63 | 29.35  49.45  19.78  1.41 |
| 7 “Unhappy – difficulty sleeping”* | 1. No, not at all 2. Not very often 3. Yes, sometimes 4. Yes, most of the time | 16.54  28.57  38.34  16.54 | 77.49  13.58  7.64  1.27 | 73.31  16.79  8.32  1.56 |
| 8.“Sad or miserable”* | 1. No, not at all 2. Not very often 3. Yes, quite often 4. Yes, most of the time | 5.63  27.81  50.37  16.16 | 55.41  36.73  7.21  0.63 | 53.53  38.46  7.53  0.47 |
| 9 “Unhappy – crying”* | 1. No, never 2. Only occasionally 3. Yes, quite often 4. Yes, most of the time | 16.91  39.84  32.33  10.90 | 63.90  32.05  3.39  0.63 | 55.10  39.40  4.55  0.94 |
| 10 “Thought of harming myself”* | 1. Never 2. Hardly ever 3. Sometimes 4. Yes, quite often | 66.16  19.54  13.15  1.12 | 96.17  3.18  0.21  0.42 | 96.86  1.88  1.25  0 |

*Reverse scoring prior to data input (the top box is scored as a 3 and the bottom box scored as 0). For the remaining questions, the top box is scored as a 0 and the bottom box scored as a 3.

**4. Factor analysis**

The McDonald’s omega (ω) internal reliability coefficients for the EPDS were good for the 10-item questionnaire for the prenatal high risk sample 0.84 (95% CI .82-.87), as well as for the 9-item questionnaire for the prenatal community sample 0.87 (95% CI .84-.89) and postnatal community sample 0.85 (95% CI .82-.87). It is important to note that these values are virtually identical to those produced by Cronbach’s α. We considered it was important to report both, because although in practice α and ω often produce similar estimates, some studies have suggested that there are certain circumstances in which the differences between them can be nontrivial (McNeish 2018).

Table S3. Suitability of data for factor analysis

|  | Largest value in corr. matrix | KMO | Bartlett’s test of sphericity | Determinant of corr. matrix |
| --- | --- | --- | --- | --- |
| **Threshold needed** | **<.90** | **>.50** | **p<.05** | **>.00001** |
| AH_EFA_Polychoric_10items | .71 | .84 | X^2^(45)=554.10  P<.001 | .004 |
| AH_EFA_Pearson_10items | .62 | .85 | X^2^(45)=376.79  p<.001 | .023 |
| AC_EFA_Polychoric_9items | .88 | .64 | X^2^(36)=975.57  p<.001 | .00006 |
| AC_EFA_Pearson_9items | .69 | .86 | X^2^(36)=784.32  P<.001 | .013 |
| PC_EFA_Polychoric_9items | .81 | .88 | X^2^(36)=610.47  P<.001 | .002 |
| PC_EFA_Pearson_9items | .67 | .88 | X^2^(36)=643.53  P<.001 | .029 |

AH = prenatal high-risk, AC=prenatal community, PC=postnatal community , < less than, > more than

**5. Additional information from main analysis**

**5.1. 3 factor models**

In all 3 samples, the 3-factor EFA revealed distinct factors for anhedonia (items 1,2 for prenatal high-risk and postnatal community, item 1 for prenatal community), anxiety (items 3,4,5), and depression (items 6,7,8,9,10 for prenatal high-risk, items 7,8,9 for prenatal community, items 8,9 for postnatal community). The models accounted for 56% of variance in the prenatal high-risk sample (anhedonia 15%, anxiety 16%, depression 25%), 61% of variance in the prenatal community sample (anhedonia 16%, anxiety 18%, depression 26%), and 58% of variance in the postnatal community sample (anhedonia 17%, anxiety 23%, depression 18%).

Anxiety and anhedonia were positively correlated in all samples, as follows: r=.51 prenatal high risk, r=.55 prenatal community, r=.65 postnatal community. Depression and anhedonia were positively correlated in all samples, as follows: r=.38 prenatal high-risk, r=.41 prenatal community, and r=.55 postnatal community. Depression and anxiety were also positively correlated in all samples: r=.44 prenatal high-risk, r=.61 prenatal community and r=.63 postnatal community.

Prenatally, the 2-factor structure differed between the high-risk sample and the community sample. In the high-risk sample, the 2-factor EFA revealed distinct anhedonia (items 1,2) and depression (items 3,5, 6,7,8,9,10) factors accounting for 51% of variance (depression 35%, anhedonia 16%), with a positive correlation between the 2 factors (r=.49). In the community sample, the 2-factor EFA did not have a clear solution; the anhedonia factor included items 1 and 2, but also 7 and 8, while the depression factor included items 3,4,5,9. This model accounted for 63% of variance (depression 27%, anhedonia 32%), with a positive correlation between the 2 factors (r=.63). Item 6 loaded on both depression and anhedonia and thus was not included in either factor. Postnatally, the 2-factor EFA revealed distinct depression (items 1,2,7,8,9) and anxiety (items 3,4,5) factors accounting for 57% of variance (depression 34%, anxiety 23%), with a positive correlation between the two factors (r=.65). Item 6 did not adequately distinguish between anxiety and depression and was not included in either factor.

*5.1.2. EFA with Pearson correlation matrix*

Table S4. EFA with Pearson Correlation Matrix

|  | Prenatal high-risk | | | | | Prenatal community | | | | | Postnatal community | | | | |
| --- | --- | --- | --- | --- | --- | --- | --- | --- | --- | --- | --- | --- | --- | --- | --- |
|  | 2-factor | | 3-factor | | | 2-factor | | 3-factor | | | 2-factor | | 3-factor | | |
|  | D | AH | D | AX | AH | D | AH | D | AX | AH | D | AX | D | AX | AH |
| 1 |  | .45 |  |  | **.50** |  | .**66** |  |  | **.65** | **.78** |  |  |  | **.73** |
| 2 |  | **.98** |  |  | **.92** |  | **1.07** |  |  | **.95** | **.64** |  |  |  | **.83** |
| 3 | .**53** |  |  | **.70** |  | **.71** |  |  | **.59** |  |  | **.61** |  | **.60** |  |
| 4 | .38 |  |  | **.68** |  | **.83** |  |  | **.90** |  |  | **.80** |  | **.88** |  |
| 5 | **54** |  |  | **.53** |  | **.67** |  |  | **.76** |  |  | **.69** |  | **.72** |  |
| 6 | **.64** |  | .43 |  |  | **.70** |  | .33 | .41 |  | **.**33 | .44 |  | .49 |  |
| 7 | **.79** |  | **.63** |  |  | .46 | .38 | **.72** |  |  | **.62** |  | .48 |  |  |
| 8 | **.64** |  | **.61** |  | .36 | **.54** |  | **.83** |  |  | **.68** |  | **.67** |  |  |
| 9 | **.69** |  | **.79** |  |  | **.72** |  | **.57** |  |  | **.64** |  | **.96** |  |  |
| 10 | .**61** |  | **.57** |  |  | --- | ---- | ---- | ---- | ---- | ---- | ---- | ---- | ---- | ---- |

D=depression factor, AH = anhedonia , AX = anxiety , substantial factor loadings (>.5) in bold

*5.1.2.1 Pearson correlation matrix – EFA for Prenatal high-risk sample*

The Kaiser criteria suggested a 3-factor model (largest eigenvalues were 4.34, 1.15, 1.13, 0.72), which was confirmed by visual inspection of scree plot. Parallel analysis conducted using MRFA suggested 3 factors.

A 2-factor model explained 44% of variance and the two factors were correlated (r=.48), but the test of the hypothesis that 2 factors are sufficient was significant X^2^(26)=45.28, p=.011, suggesting that a model with a higher number of factors may be more appropriate. A three factor model explained 47% of variance, X^2^(18)=14.34, p=.707.

*5.1.2.2. Pearson correlation matrix – EFA for Prenatal community sample*

The Kaiser criteria suggested a 2-factor model (largest eigenvalues were 4.62, 1.12, 0.75), which was confirmed by visual inspection of the scree plot. Parallel analysis conducted using MRFA suggested 3 factors.

A 2-factor model explained 56% of variance and the 2 factors were correlated (r=.65), but the test of the hypothesis that 2 factors are sufficient was significant X^2^(19)=60.24, p<.001, suggesting that a model with a higher number of factors may be more appropriate. A 3-factor model explained 57% of variance, with X^2^(12)=22.17, p=.035. Item 6 did not adequately distinguish between Factor 1 and 2.

*5.1.2.3. Pearson correlation matrix – EFA for Postnatal community sample*

The Kaiser criteria suggested a 2-factor model (largest eigenvalues were 4.32, 1.16, 0.76), which was confirmed by visual inspection of the scree plot. Parallel analysis conducted using MRFA suggested 3 factors.

A 2-factor model explained 46% of variance and the 2 factors were correlated (r=.65), but the test of the hypothesis that 2 factors are sufficient was significant X^2^(19)=55.74, p<.001, suggesting that a model with a higher number of factors may be more appropriate. A 3-factor model explained 55% of variance, with X^2^(12)=14.44, p=.274.

*5.1.3. CFA using MLE*

| Table S5. CFA using MLE | | | | | | | | | | | | |
| --- | --- | --- | --- | --- | --- | --- | --- | --- | --- | --- | --- | --- |
| Model tested | Sample |  | | |  | | | Fit indices | | | | |
|  |  | X^2^ | P* | df | | CFI | TLI | | RMSEA  (LO90, HI90) | AIC | BIC | SRMSR |
| **EFA 3 factors**  AH (1,2), AX (3,4,5), D (7-10)  AH (1), AX (3,4,5), D (7-8)  AH (1,2), AX (3,4,5), D (8,9) | High-risk A.  Comm A.  Comm P. | 28.30  10.6618.38 | .248  .154  .073 | 36  7  11 | | .989  .994  .992 | .984  .986  .985 | | .033 (.075-.695)  .043(.000-.092)  .042(.000-.074) | 3064.0  2772.3  4537.1 | 3128.6  2823.3  4604.2 | .047  .027  .024 |
| **EFA 2 factors**  AH (1,2), D(3,5-10)  AH (1,2,7,8), D (3,4,5,9)  D (1,2, 7-9), AX (3,4,5) | High-risk A.  Comm A.  Comm P. | 35.68  37.04  83.93 | .098  <.001  <.001 | 26  13  28 | | .975  .965  .938 | .966  .943  .909 | | .048(.000-.084)  .081(.051-.112)  .095(.074-.116) | 3019.5  2935.9  5272.9 | 3077.9  2990.6  5340.0 | .046  .045  .047 |
| **One factor** | High-risk A.  Comm A.  Comm P. | 88.95  153.1  185.1 | <.001  <.001  <.001 | 35  27  27 | | .881  .891  .871 | .847  .854  .828 | | .098(.073-.124)  .129(.109-.149)  .124(.107-.141) | 3366.8  3704.5  6020.1 | 3428.3  3770.1  6091.1 | .071  .067  .068 |
| **Phillips 2 factors**  D (1,2,6-10), AX (3,4,5) Same-EFA  D (1,2,6-9), AX (3,4,5)  D (1,2, 6-9), AX (3,4,5) | High-risk A.  Comm A.  Comm P. | -  109.0  103.3 | -  <.001  <.001 | -  26  26 | | -  .928  .937 | -  .900  .913 | | -  .106(.086-.127)  .088(.071-.106) | -  3662.4  5940.2 | -  3731.6  6015.2 | -  .056  .050 |
| **Zhong 2 factors**  AH (1,2), D (3-10)  AH (1,2), D (3-9)  AH (1,2), D (3-9) | High-risk A.  Comm A.  Comm P. | 72.19  97.32  122.0 | <.001  <.001  <.001 | 34  26  26 | | .916  .938  .922 | .889  .914  .892 | | .084(.057-.111)  .098(.078-.120)  .098(.081-.116) | 3352.1  3650.7  5959.0 | 3416.6  3719.9  6034.0 | .064  .055  .055 |
| **Brouwers 2-3 factors**  D (1,2,8), AX (3,4,5), SH (10)  D (1,2,8), AX (3,4,5)  D (1,2,8), AX (3,4,5) | High-risk A.  Comm A.  Comm P. | 22.34  47.36  45.59 | .034  <.001  <.001 | 12  8  8 | | .959  .934  .942 | .928  .876  .892 | | .073(.020-.120)  .132(.097-.169)  .111(.081-.143) | 2376.1  2494.7  4089.8 | 2425.3  2542.1  4141.1 | .061  .060  .058 |
| **Lau 3 factors**  AH (1,2), AX (3,4,5), D(6-10)  AH (1,2), AX (3,4,5), D(6-9)  AH (1,2), AX (3,4,5), D(6-9) | High-risk A.  Comm A.  Comm P. | 38.31  55.79  44.90 | .205  <.001  .006 | 32  24  24 | | .986  .972  .983 | .980  .959  .973 | | .035(.000-.071)  .068(.045-.092)  .048(.025-.069) | 3322.2  3613.1  5885.8 | 3392.9  3689.7  5968.7 | .049  .039  .033 |
| **Tuohy 3 factors**  AH (1,2),AX (3,4,5),D(7-10)  Same as EFA  AH (1,2), AX (3,4,5), D(7-9)  AH (1,2), AX (3,4,5), D(7-9) | High-risk A.  Comm A.  Comm P.. | -  37.25  28.86 | -  .003  .036 | -  17  17 | | -  .980  .989 | -  .967  .981 | | -  .065(.036-.093)  .043(.011-.069) | -  3125.2  5221.9 | -  3194.5  5296.8 | -  .033  .028 |
| **Kubota 3 factors**  AH (1,2), AX (3,4,5), D(7-9)  AH (1,2), AX (3,4,5), D(7-9)- Tuohy  AH (1,2), AX (3,4,5), D(7-9)-Tuohy | High-risk A.  Comm A.  Comm P. | 24.15  -  - | .115  -  - | 17  -  - | | .981  -  - | .968  -  - | | .051(.000-.095)  -  - | 2709.2  -  - | 2767.6  -  - | .048  -  - |

*is sensitive to sample size, and can be significant for large samples, ^ model is not identified A=Prenatal, P=Postnatal, D=depression,, AX=anxiety, AH=anhedonia, SH=self-harm . Where MLE was performed, we also used the Akaike Information Criterion (AIC) and Bayesian Information Criterion (BIC) to compare models (smaller values are better).

*5.1.4. Whole-sample EFA*

We conducted an EFA on the whole sample (n=1190), to examine whether there is a common factor structure for women in the perinatal period. This sample included all high-risk participants (from Perinatal Stress study) and one timepoint (randomly selected) for each dHCP participant. The internal reliability coefficient for the EPDS was good; the values for both Cronbach’s α and McDonald’s ω were 0.92(95% CI .91-.92). The dataset met all criteria for factor analysis, as follows: largest value in correlation matrix <.90, KMO =.95,

Bartlett’s test of sphericity X^2^ (45)=1101.29, p<.001, determinant of correlation matrix =.00008. The scree plot and parallel analysis suggested that the most appropriate model would contain 2 or 3 factors.

A 2-factor model revealed separate depression (items 1,2,6,7,8,9) and anxiety (items 3,4,5) factors, accounting for 62% of variance (39% and 23% respectively). The two factors were strongly correlated (r=.81). Item 10 did not adequately distinguish between the 2 factors.

The structure of the 3 factor model (accounting for 57% of variance) was not as clear. This model revealed separate depression (Item 9, accounting for 15% of variance), anxiety (Items 3,4,5, accounting for 22% of variance), and anhedonia factors (Items 1,2, accounting for 20% of variance). Several items loaded on multiple factors. Item 10 loaded on both anxiety and depression, while items 7 and 8 loaded on both depression and anhedonia. Item 6 did not substantially (>.5) load on any factors (.37 on anhedonia). The factors correlated as follows: r=.76 for anxiety and anhedonia, r=-.80 for anxiety and depression, r=-.76 for anhedonia and depression.

Table S6. Whole sample EFA

|  | Whole sample | | | | |
| --- | --- | --- | --- | --- | --- |
|  | 2-factor | | 3-factor | | |
|  | D | AX | D | AX | AH |
| 1 | **1.00** |  |  |  | **.91** |
| 2 | **.92** |  |  |  | **.89** |
| 3 |  | **.61** |  | **.58** |  |
| 4 |  | **.79** |  | **.85** |  |
| 5 |  | **.93** |  | **.91** |  |
| 6 | **.62** |  |  |  | .37 |
| 7 | **.68** |  | .40 |  | .32 |
| 8 | **.81** |  | .49 |  | .39 |
| 9 | **.68** |  | **.88** |  |  |
| 10 | .35 | .49 | .41 | .43 |  |

substantial factor loadings (>.5) in bold

**6. EPDS cut-off scores**

For the EPDS total score, the original EPDS study (Cox et al. 1987) suggested cut-offs of 10 or 13, with studies using values that range between these two numbers, and 13 being the most common cut-off (Milgrom & Gemmill 2015). However, a recent meta-analysis suggested that a cut-off value of 11 or higher maximised combined sensitivity and specificity across reference standards (Levis et al. 2020). Based on this recent evidence, this study uses a cut-off of 11 or higher, but we also report values for a cut-off of 13, for consistency with previous literature.

For the EPDS-3A, we present our data using two different cut-offs for the EPDS-3A, as there is currently no agreed-upon validated cut-off for this subscale in prenatal and postnatal women in the UK. The 4 or more cut-off was validated in a postnatal sample of Australian women with unsettled infants (Phillips et al. 2009), while the 6 or more cut-off was validated in a community postnatal sample of Australian women (Matthey et al. 2009). We advise caution when interpreting our findings, as the proportion of women with probable anxiety symptoms in our sample is markedly different depending on the cut-off that is used. While a lower cut-off may be helpful for initial screening in clinical settings, a higher cut-off may be more appropriate in research settings, where minimising false-positives is important (Phillips et al. 2009).

In our sample, we aimed to assess the number of women that may have anxiety symptoms (based on EPDS-3A) but do not score high enough on the EPDS total to warrant further assessment. We are reporting these numbers for various cut-offs (Table S7)

Table S7. Number of women scoring high on anxiety symptoms but low on depression symptoms using different cut-offs

| EPDS total score cut-off | EPDS-3A cutoff | Prenatal high risk  N (%) | Prenatal community  N (%) | Postnatal community  N (%) |
| --- | --- | --- | --- | --- |
| 10 or less | 4 or more | 27 (10.15%) | 102 (21.56%) | 142 (22.18%) |
| 10 or less | 6 or more | 9 (3.38%) | 9 (1.90%) | 16 (2.5%) |
| 12 or less | 4 or more | 55 (20.67%) | 125 (26.42%) | 167 (26.09%) |
| 12 or less | 6 or more | 21 (7.89%) | 21 (4.43%) | 30 (4.68%) |

These discrepancies highlight the urgent need for agreed upon cut-offs that are used throughout clinical settings.

Pending further validation studies, using the EPDS-3A in conjunction with the EPDS total score would facilitate patient-provider communication around anxiety symptoms, and could lead to interventions that would positively impact the health of mother and baby.

**7. Relationship with anxiety measures**

In a sub-sample of n=24 high-risk participants, with high EPDS total scores (mean=17.54, SD=4.06, range 13-25), data were available for State Trait Anxiety Inventory, Prenatal Distress Questionnaire (NUPDQ), Perceived Stress Scale (PSS). No formal analysis was done due to the small sample size, but visual inspection of plots suggests that there may be a positive correlation between EPDS-3A scores and scores on State-Trait Anxiety Inventory, as well as Prenatal Distress (Figure S3). We advise caution in interpreting these exploratory findings and call for further research to investigate these relationships in a larger sample.

Fig S3. Relationship between anxiety measures and EPDS-3A in a high-risk subsample


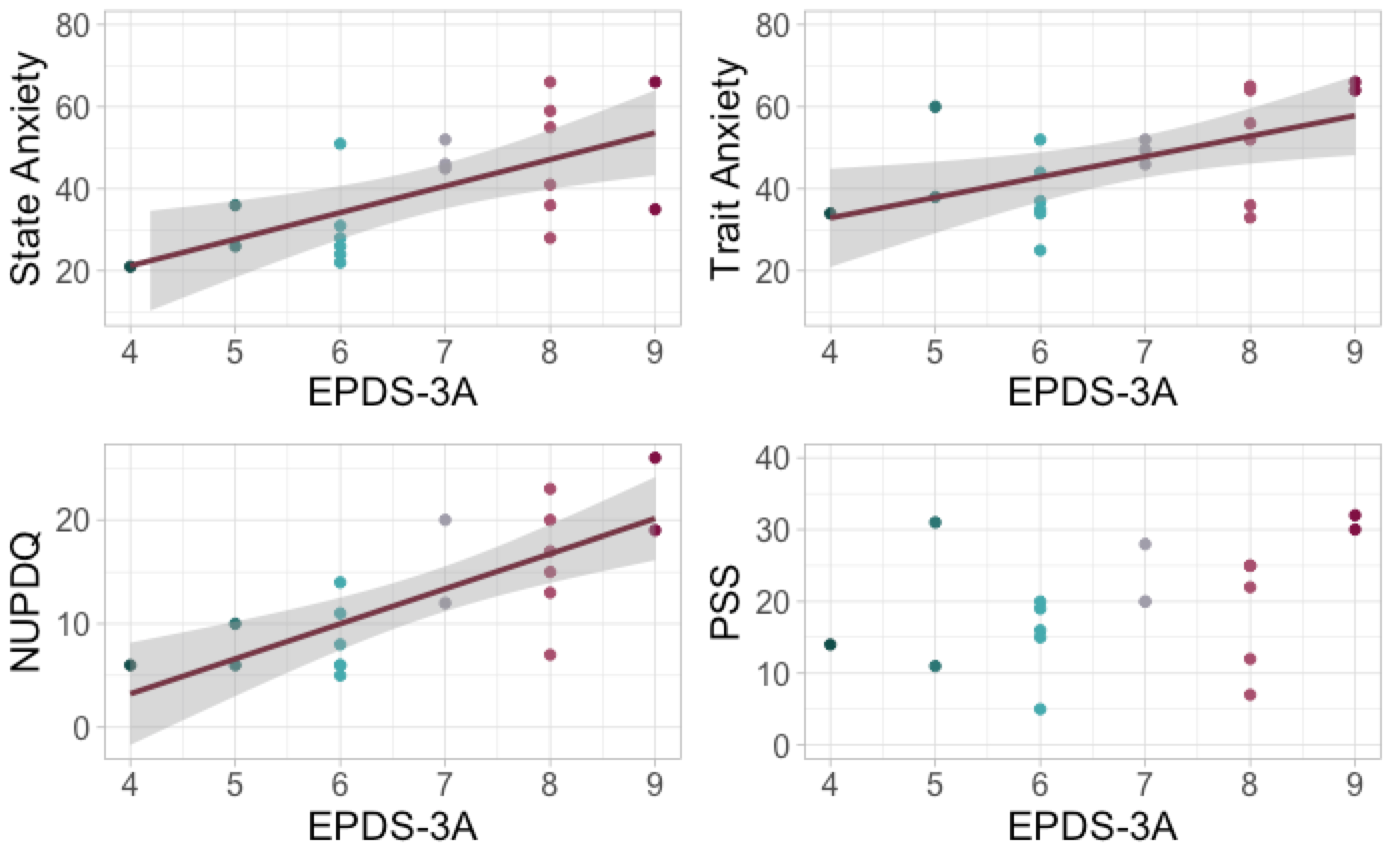


**8. EPDS and history of mental health concerns**

In the literature, perinatal depression is not only associated with a history of depression, but also with a history of poor mental health (Thomson et al. 2020). For example, mothers who disclose a history of poor mental health are 4 times more likely to score high on the EPDS (Dennis and Ross 2009). With this in mind, we examined the relationship between EPDS total score and a history of mental health conditions.

In the main manuscript, we reported that EPDS total score was higher in those with a history of mental health conditions than those without a history. The “History of mental health concerns” variable was a binary variable coded yes/no, with “Yes” indicating either a specific mental health condition noted in the mother’s medical notes, or a “Yes” on a self-report question (e.g. “Have you ever been treated for mental health”).

We further examined the distribution of EPDS total scores in participants who reported a history of depression and/or anxiety specifically.

In the prenatal community sample, data suggested that n=48 participants had a history of depression but not anxiety (Depression only), n=26 had a history of anxiety but not depression (Anxiety only), n=32 had a history of both depression and anxiety (Both), and n=367 had no history of either (Neither). In the postnatal community sample, data suggested that n=72 participants had a history of depression but not anxiety (Depression only), n=19 had a history of anxiety but not depression (Anxiety only), n=32 had a history of both depression and anxiety (Both), and n=516 had no history of either (Neither). We recommend that further studies examining this relationship aim for equal group sizes. However, in this study, we report descriptive statistics for these subgroups, for exploratory purposes only.

Prenatally, the mean EPDS total score was similar in the “Depression only” group (mean=10.12, SD=6.06) and “Both” group (mean=10.81, SD=7.91), slightly lower in the “Anxiety only” group (mean=8.53, SD=5.21), and much lower in the “Neither” group (mean=4.12, SD=3.35).

Fig S4. Raincloud plots showing distribution of the highest prenatal EPDS total scores in women with a history of depression but not anxiety (“Depression only”), anxiety but not depression (“Anxiety only”), both anxiety and depression (“Both”), and neither anxiety nor depression (“Neither”). For each group, jittered raw data are shown on the left, boxplots with median and interquartile range are shown in the middle, and density plots are shown on the right.


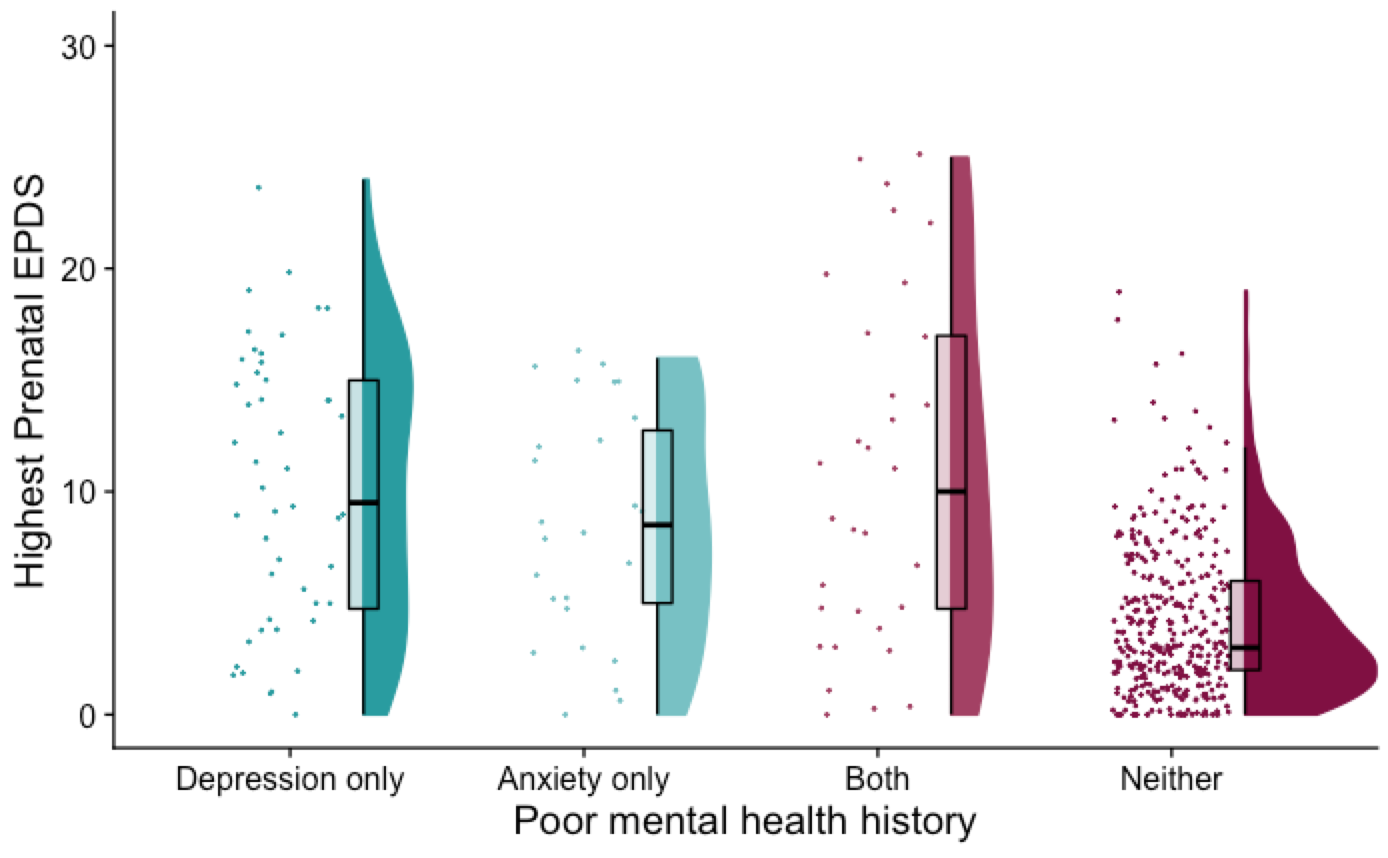


Postnatally, the mean EPDS total score was similar in the “Depression only” group (mean=7.95, SD=5.74) and “Both” group (mean=7.21, SD=4.68), slightly lower in the “Anxiety only” group (mean=6.31, SD=4.38), and lower in the “Neither” group (mean=5.16, SD=4.03).

Fig S5. Raincloud plots showing distribution of the highest postnatal EPDS total scores in women with a history of depression but not anxiety (“Depression only”), anxiety but not depression (“Anxiety only”), both anxiety and depression (“Both”), and neither anxiety nor depression (“Neither”). For each group, jittered raw data are shown on the left, boxplots with median and interquartile range are shown in the middle, and density plots are shown on the right.


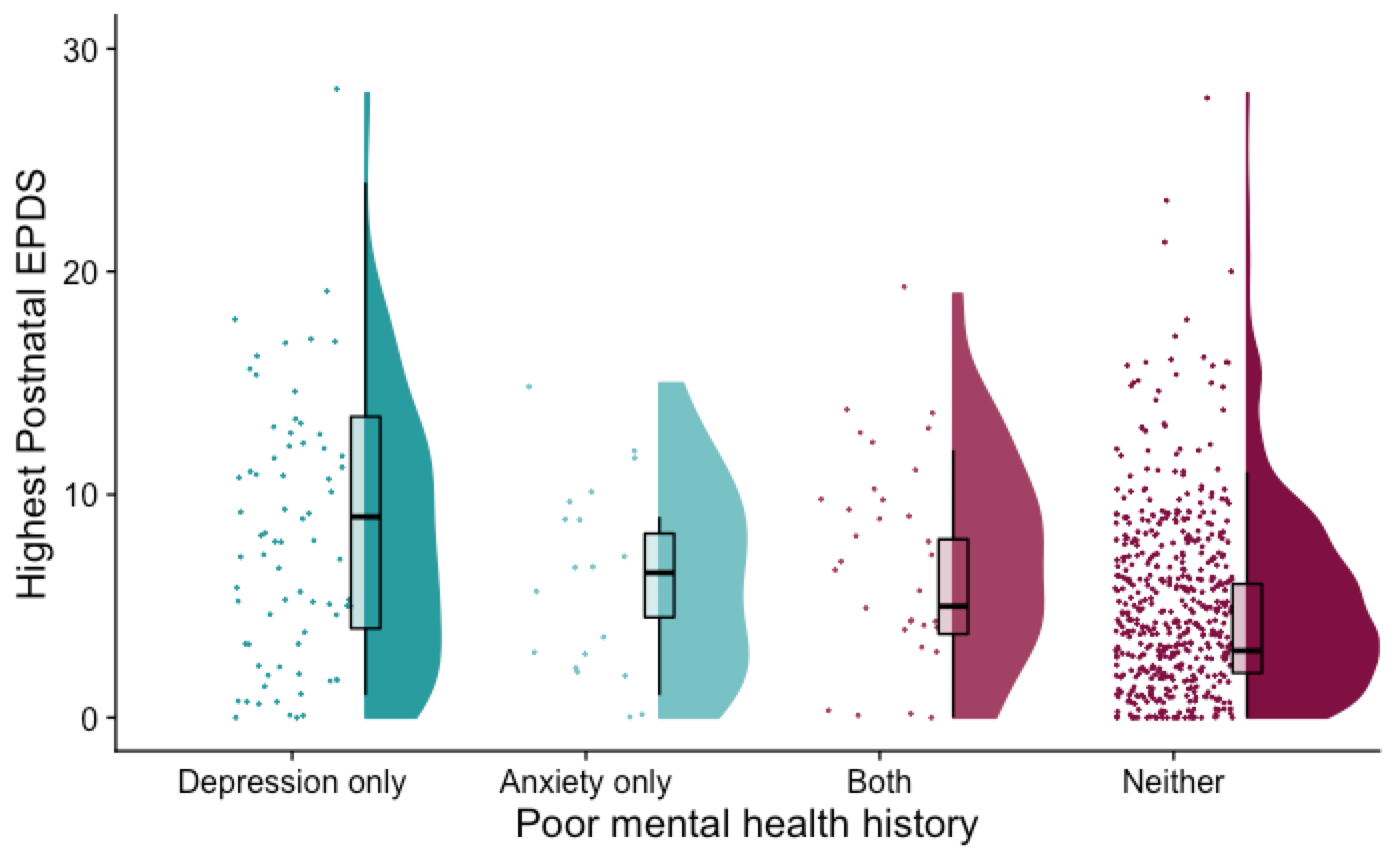


In addition, we report the number of participants in each group based on their total EPDS score and history of mental health (Table S8).

Table S8. Number of participants scoring above threshold on the EPDS based on their history of poor mental health

| Sample | Highest total EPDS | History | N |
| --- | --- | --- | --- |
| Prenatal | 11 or more | Poor mental health | 55 |
|  | 11 or more | No evidence of poor mental health | 13 |
|  | 10 or less | Poor mental health | 93 |
|  | 10 or less | No evidence of poor mental health | 312 |
| Postnatal | 11 or more | Poor mental health | 41 |
|  | 11 or more | No evidence of poor mental health | 37 |
|  | 10 or less | Poor mental health | 126 |
|  | 10 or less | No evidence of poor mental health | 437 |

**9. EPDS-3A and history of mental health concerns**

Prenatally, the mean EPDS-3A was similar in the “Depression only” group (mean=4.43, SD=2.32), “Anxiety only” group (mean=4.19, SD=2.31), and “Both” group (mean=4.53, SD=2.62), but lower in the “Neither” group (mean=2.33, SD=1.74).

Fig S6. Raincloud plots showing distribution of the highest prenatal EPDS-3A scores in women with a history of depression but not anxiety (“Depression only”), anxiety but not depression (“Anxiety only”), both anxiety and depression (“Both”), and neither anxiety nor depression (“Neither”). For each group, jittered raw data are shown on the left, boxplots with median and interquartile range are shown in the middle, and density plots are shown on the right.


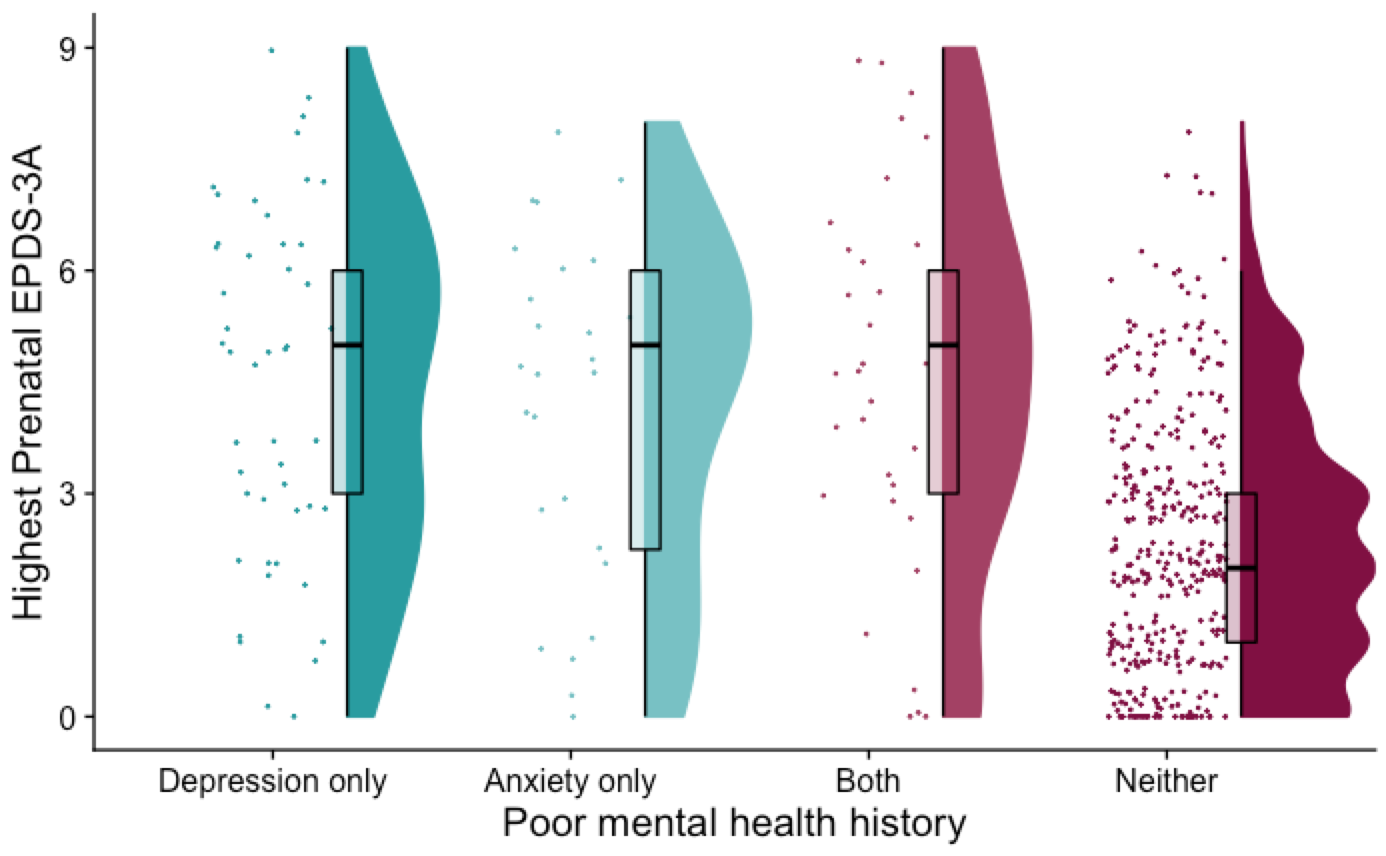


Postnatally, the mean EPDS-3A was similar in the “Depression only” group (mean=3.47, SD=2.29), “Anxiety only” group (mean=3.15, SD=2.33), and “Both” group (mean=3.31, SD=2.30), but lower in the “Neither” group (mean=2.57, SD=1.88).

Fig S7. Raincloud plots showing distribution of the highest prenatal EPDS-3A scores in women with a history of depression but not anxiety (“Depression only”), anxiety but not depression (“Anxiety only”), both anxiety and depression (“Both”), and neither anxiety nor depression (“Neither”). For each group, jittered raw data are shown on the left, boxplots with median and interquartile range are shown in the middle, and density plots are shown on the right.


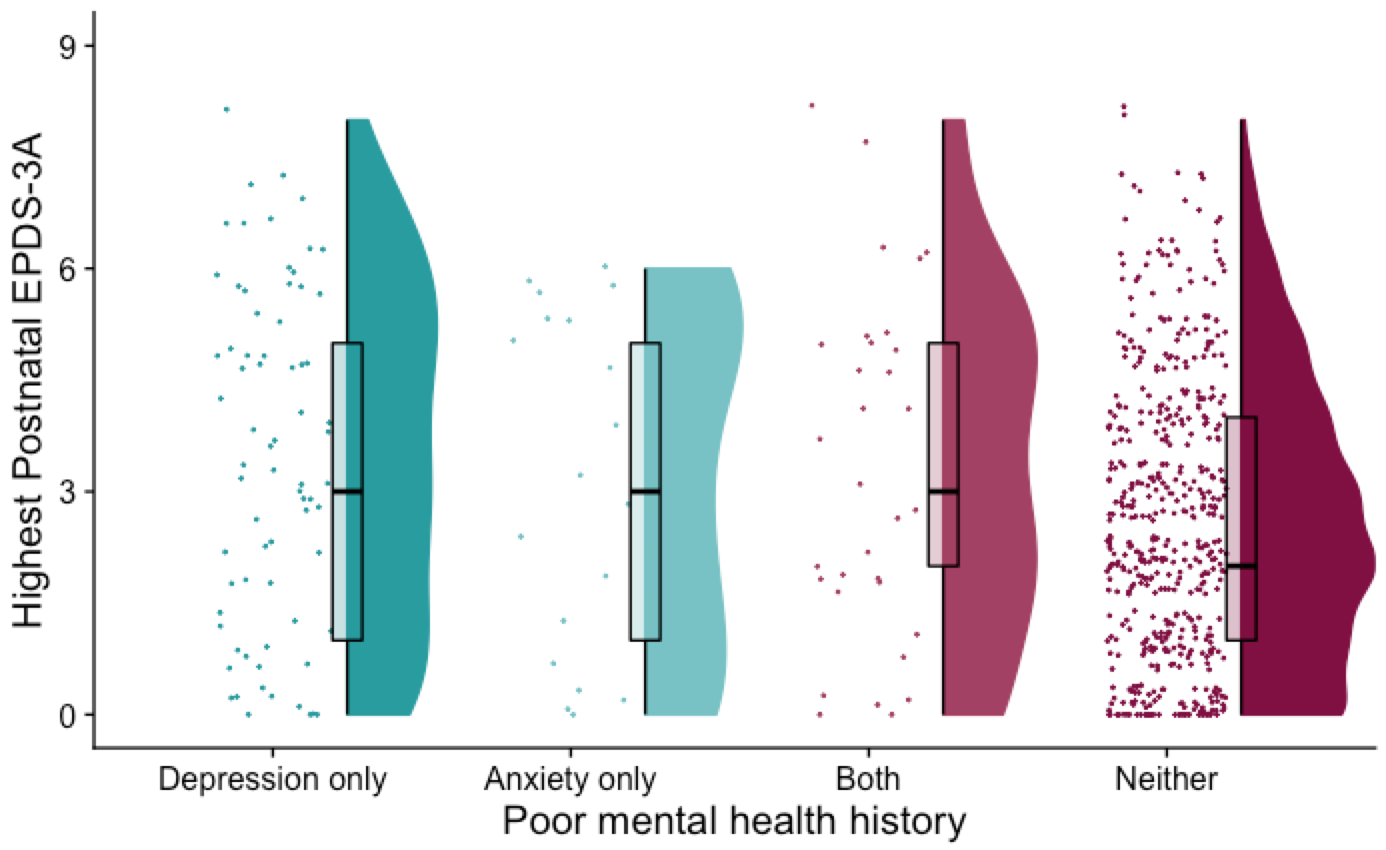


However, it is important to note that information regarding maternal history of mental health concerns was collected from secondary sources, and did not include information regarding diagnoses during pregnancy. Thus, further research is required to determine whether the EPDS 3A uniquely relates to anxiety disorders relative to other mental health concerns.

As maternal anxiety and depression are often comorbid, it is difficult to appropriately distinguish between those with a history of depression (but not anxiety) and those with a history of anxiety (but not depression). This is of particular importance given recent evidence suggesting that anxiety and depression may have independent effects on the developing fetal brain (e.g. Reissland et al. 2018).

In addition, we report the number of participants in each group based on their EPDS-3A score and history of anxiety (Table S9).

Table S9. Number of participants scoring above threshold on the EPDS-3A based on their history of anxiety

| Sample | Highest EPDS-3A | History | N |
| --- | --- | --- | --- |
| Prenatal | 4 or more | History of anxiety | 38 |
|  | 4 or more | No history of anxiety | 116 |
|  | 3 or less | History of anxiety | 20 |
|  | 3 or less | No history of anxiety | 299 |
| Postnatal | 4 or more | History of anxiety | 24 |
|  | 4 or more | No history of anxiety | 192 |
|  | 3 or less | History of anxiety | 27 |
|  | 3 or less | No history of anxiety | 398 |

**10. Vargha and Delaney’s A effect size measure**

To determine the effect size, we used Vargha and Delaney’s effect size (vd.a) measure as calculated by the effsize package in R. Vd.a represents the probability that an observation in one group is larger than an observation in the other group, with vd.a (A,B) = 1 – vd.a (B, A). The effsize package provides not only the value, but also a qualitative assessment of the magnitude of effect size, and we have provided this qualitative assessment in brackets following each effect size. For reference, when interpreting vd.a, a value of .50 would represent no effect, while values of 0 or 1 represent complete stochastic dominance of one group over the other.

Large effect sizes are ≥.71 or ≤.29, medium effect sizes are .64 to <.71 or >.29 to .34, and small effect sizes are .56 to <.64 and >.34 to .44 (Vargha and Delaney 2000).

**11. Preterm birth**

The community postnatal sample contains a number of babies born prematurely. We considered it was important to report the distribution of maternal EPDS total scores based on gestational age at birth in this sample. Mothers of babies that were born extremely preterm (GA < 28 weeks, n=29) had higher EPDS total scores (mean=10.13 ± 5.36, range 2-23) compared to the other subgroups. Mothers of babies born very preterm (GA 28.0 -31.9 weeks, n=43) scored 5.32 ± 4.50 (range 0-20), while mothers of babies born moderate to late preterm (GA 32.0 -36.9 weeks, n=78) scored 6.20 ± 4.61 (range 0-21). The rest of the sample was composed of babies born at term (GA 37 weeks and over), and the mean EPDS total score was 5.22 ± 4.12.

The distribution of scores was similar for the EPDS-3A. Mothers of babies that were born extremely preterm had higher EPDS-3A scores (mean=4 ± 2.35, range 0-7) compared to mothers of babies born very preterm (mean = 2.27 ± 2.03, range=0-8), moderate to late preterm (mean=3.05, range=2.05, range=0-8) or at term (mean=2.63 ± 1.91, range 0-8).

Further research in larger samples is required to investigate whether the factor structure of the EPDS differs between these groups, as it is likely that both EPDS total and EPDS-3A scores are influenced by the stress-inducing situation of having a preterm baby. A better understanding of the EPDS factor structure in this already vulnerable group is of particular importance given recent evidence that maternal prenatal stress impacts brain development in babies born prematurely ( Scheinost et al. 2016, Lautarescu et al. 2020).

**References**

Dennis CL & Ross LE (2006) The clinical utility of maternal self-reported personal and familial psychiatric history in identifying women at risk for postpartum depression. Acta Obstetricia et Gynecologica Scandinavica 85(10), 1179-1185. <https://doi.org/10.1080/00016340600697595>

Lautarescu A, Pecheva D, Nosarti C, Nihouarn J, Zhang H, Victor S, ... & Counsell SJ (2020) Maternal prenatal stress is associated with altered uncinate fasciculus microstructure in premature neonates. Biological Psychiatry 87(6), 559-569. <https://doi.org/10.1016/j.biopsych.2019.08.010>

Matthey S (2008) Using the Edinburgh Postnatal Depression Scale to screen for anxiety disorders. Depression and Anxiety 25, 926–931. <https://doi.org/10.1002/da.20415>

McNeish D (2018) Thanks coefficient alpha, we’ll take it from here. Psychological Methods 23(3), 412. [https://doi.org/10.1037/met0000144](https://psycnet.apa.org/doi/10.1037/met0000144)

Phillips J, Charles M, Sharpe L, & Matthey S (2009) Validation of the subscales of the Edinburgh Postnatal Depression Scale in a sample of women with unsettled infants. Journal of Affective Disorders 118, 101–112. <https://doi.org/10.1016/j.jad.2009.02.004>

Reissland N, Froggatt S, Reames E, & Girkin J (2018) Effects of maternal anxiety and depression on fetal neuro-development. Journal of Affective Disorders 241, 469-474. <https://doi.org/10.1016/j.jad.2018.08.047>

Scheinost D, Kwon SH, Lacadie C, Sze G, Sinha R, Constable RT, & Ment LR (2016) Prenatal stress alters amygdala functional connectivity in preterm neonates. NeuroImage: Clinical 12, 381-388. <https://doi.org/10.1016/j.nicl.2016.08.010>

Thomson KC, Romaniuk H, Greenwood CJ, Letcher P, Spry E, Macdonald JA, ... & Olsson CA (2020) Adolescent antecedents of maternal and paternal perinatal depression: a 36-year prospective cohort. Psychological medicine 1-8.  <https://doi.org/10.1017/S0033291720000902>

Vargha A & Delaney HD (2000) A critique and improvement of the CL common language effect size statistics of McGraw and Wong. Journal of Educational and Behavioral Statistics 25(2), 101-132. [https://doi.org/10.3102/10769986025002101](https://doi.org/10.3102%2F10769986025002101)
